# Patient‐specific mapping of fundus photographs to three‐dimensional ocular imaging

**DOI:** 10.1002/mp.17576

**Published:** 2024-12-12

**Authors:** Corné Haasjes, T. H. Khanh Vu, Jan‐Willem M. Beenakker

**Affiliations:** ^1^ Department of Ophthalmology Leiden University Medical Center Leiden The Netherlands; ^2^ Department of Radiology Leiden University Medical Center Leiden The Netherlands; ^3^ Department of Radiation Oncology Leiden University Medical Center Leiden The Netherlands

**Keywords:** fundoscopy, optics, uveal melanoma

## Abstract

**Background:**

Ocular proton beam therapy (OPT) planning would benefit from an accurate incorporation of fundus photographs, as various intra‐ocular structures, such as the fovea, are not visible on conventional modalities such as Magnetic Resonance Imaging (MRI). However, the use of fundus photographs in OPT is limited, as the eye's optics induce a nonuniform patient‐specific deformation to the images.

**Purpose:**

To develop a method to accurately map fundus photographs to three‐dimensional images.

**Methods:**

Personalized optical raytracing simulations were performed for 27 subjects, using subject‐specific eye models based on corneal topography, biometry, and MRI. Light rays were traced through the eye for angles of 0°–85° with respect to the optical axis, in steps of 5°. These simulations provided a reference mapping between camera angles and retinal locations and were used to develop a mapping method without raytracing. The accuracy of this and earlier proposed methods was evaluated. Finally, the most accurate method was implemented in RayOcular, an image‐based OPT planning system, and the fundus photography‐based tumor contour was compared with MRI.

**Results:**

When a patient‐specific second nodal point is taken as a reference to describe the retinal location, the camera, and retinal angles show a strong linear relation with a small variation between subjects. At a camera angle of 60°, for example, a corresponding retinal angle of 59.9° ± 0.4° (mean ± SD) was found. When this linear relation is used to predict the corresponding retinal location (without raytracing) of a camera angle of 40°, the mean (Euclidian distance) error in the retinal location was 0.02 mm (SD = 0.06 mm), which was significantly (*p* < 0.001) lower than earlier proposed methods including EYEPLAN 4.16 mm (SD = 0.25 mm), the Lamberth projection −0.12 mm (SD = 0.46 mm) or polar projection 0.26 mm (SD = 0.57 mm). When implemented in the fundus view of RayOcular, the median distance between contours based on MRI and fundus photography was 0.2 mm (IQR = 0.1–0.3 mm).

**Conclusions:**

The second nodal point provides a patient‐specific reference for an accurate mapping of fundus photographs to three‐dimensional images with sub‐millimeter errors.

## 1 INTRODUCTION

In ocular proton therapy planning multiple sources of information, including ultrasound and intraoperatively measured tumor‐marker distances, are combined to create a three‐dimensional geometrical eye and tumor model on which the treatment planning is performed.^1^, ^2^, ^3^ Although high rates of local control have been achieved with such model‐based planning, a generic mathematical model does not fully describe the actual shape of the tumor. To compensate for uncertainties in the combination of different modalities, this model overestimates the target volume.^4^, ^5^ Magnetic Resonance Imaging (MRI) has been proposed as an alternative input for defining the tumor volume, as it allows for a three‐dimensional evaluation of the tumor and surrounding anatomy with sub‐millimeter accuracy.^6^, ^7^, ^8^, ^9^, ^10^, ^11^ However, as the fovea, the part of the retina responsible for sharp vision, cannot be identified on MRI, and flat tumor extensions cannot be reliably delineated on MRI,^4^, ^10^, ^12^ it is desirable to combine optical fundus images with three‐dimensional imaging.

Accurately correlating locations on fundus photographs to their actual location in three‐dimensional space is; however, nontrivial as both the optics of the eye and the retinal geometry vary from subject to subject.^1^, ^12^, ^13^, ^14^, ^15^ The scaling of a fundus photograph, for example, depends strongly on subject‐specific optical parameters, primarily the curvature of the cornea and distances of the cornea and eye lens to the retina.^16^, ^17^ The impact of these differences has recently been shown by Pors et al., who found a more than 30% scaling difference at the central part of fundus images between mildly myopic and hyperopic subjects, regardless of refractive state.^17^ These optical effects become even more complex when a larger part of the retina is evaluated, as peripheral light rays are strongly refracted at the optical interfaces of the eye, that is the cornea and lens front and back surfaces.^18^ Furthermore, the retina is a curved surface, which is projected onto a two‐dimensional plane. Similar to topographic maps, this projection results in a nonuniform image deformation, hindering accurate distance measurements.^19^ In the past, different methods have been proposed to combine information in fundus images with 3D treatment planning data. However, as these methods do not consider the optics of the eye, additional information, such as fiducials stitched to the eye wall, is currently needed to obtain a good match.^12^, ^13^, ^14^, ^15^, ^20^

In the last years, computationally assisted optical raytracing methods have become available, which enable optical simulations of the eye on a subject‐specific level. These methods are now commonly used in refractive surgery to determine the optimal intra‐ocular lens (IOL) power for cataract surgery,^21^, ^22^, ^23^, ^24^ but also to obtain insight into optics‐related visual conditions such as negative dysphotopsia.^25^, ^26^, ^27^ In these raytracing simulations, the relevant elements of the ocular geometry are personalized and the exact path of the light rays through the eye can be obtained to, for example, calculate a subject's refraction,^26^ or determine the extent of the shadow cast by an intra‐ocular tumor during marker surgery.^4^

In this study, we aim to employ these patient‐specific raytracing methods to determine the actual relation between retinal and fundus photograph locations. Based on this “ground‐truth” mapping, we will develop a method to correlate locations on fundus photographs to the 3D ocular geometry without raytracing and compare this method with the previously proposed approaches.

## 2 MATERIALS AND METHODS

The central part of this study is a set of extensive optical simulations with 27 subject‐specific eye models. We will therefore first describe the general setup of the optical simulations, followed by details on how the eye was modeled to match the subject's anatomy. Subsequently, the results of these simulations will be used to assess the relation between locations on fundus images and locations on the retina. Based on these relations, we will develop a mapping between fundus image locations and the corresponding retinal locations without raytracing. This method will be compared with earlier proposed methods to include fundus photos in ocular radiotherapy planning.^12^, ^13^, ^14^, ^15^ Finally, we will provide a proof‐of‐concept by integrating the developed method into an ocular treatment planning system.

**FIGURE 1:**
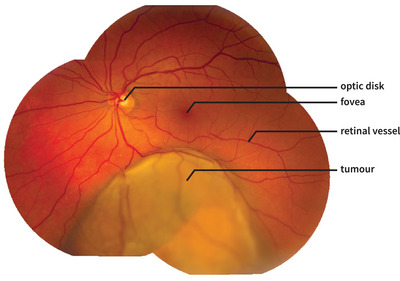
Stitched fundus photograph of a patient with uveal melanoma. This photograph is created by stitching together multiple fundus photographs that have been acquired with a classical fundus camera (Topcon TRC‐50DX, Topcon KK, Tokyo, Japan). Various retinal structures, such as the fovea and retinal vessels, are visible on fundus photographs but not on MR images. The tumor is approximately 10 mm wide.

**FIGURE 2:**
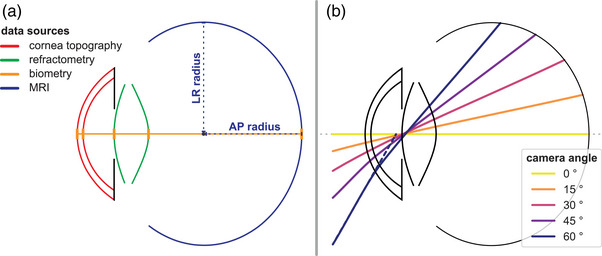
(a) Schematic representation of the eye model used for optical raytracing simulations. The model combines optical and geometrical data from multiple sources. The retinal shape is obtained from an ellipsoidal fit on MRI data. (b) Example ray trace results for various camera angles, measured with respect to the optical axis. Input rays are aimed at the paraxial entrance pupil (dashed line at 60°), which is located slightly anterior to the real pupil location. MRI, magnetic resonance imaging.

Optically, fundus photography involves two distinct optical systems, the eye and the fundus camera. In this study, we will consider an “ideal” fundus camera, in the sense that the whole image is in focus. Hence, a single light ray emitted from the retina to the camera is sufficient to describe the relation between the retina and the image plane. This assumption basically describes a stitched mosaic fundus photograph when each individual photo only describes a small area and thus is in focus (Figure 1). Under this condition, the location on the fundus photograph can be expressed as the angle of the chief light ray leaving the eye through the entrance pupil with respect to the visual axis (Figure 2b).^28^ ^(p. 43)^

Optical raytracing simulations were performed in OpticStudio (version 23 R2, ANSYS, Inc.) using ZOSPy 1.1.1 with a wavelength of 543 nm.^29^ For each of the 27 subjects a personalized eye model was made based on corneal topography (Pentacam OCULUS, Optikgerate GmbH, software version 1.20r41), biometry (Lenstar LS900, Haag‐Streit AG), refractometry and high‐resolution MRI (Achieva 7 Tesla, Philips, Best, The Netherlands)^21^, ^22^, ^23^, ^24^ (Table 1). These data have been acquired earlier in the context of a study for which the subjects, with no known ophthalmic history, have provided written informed consent.^25^, ^26^, ^30^, ^31^ The cornea curvature and asphericity were based on the six central millimeters of the cornea, while the on‐axis positions of all surfaces were obtained from biometry. The lens back curvature and asphericity were fitted to match the patient's spherical equivalent of refraction.^17^ The retina was modeled as an elliptic surface, with the half‐axes obtained from an ellipsoidal fit on T1‐weighted ocular MRI scans with a resolution of 0.5 × 0.5 × 1.0 mm^3^, as detailed by Beenakker et al.^30^, ^31^ In brief, after preprocessing of the MR images, the retinal and lens contours were automatically determined with sub‐voxel precision by fitting a mesh to the gradient of the images. Subsequently, the gazing direction was defined as the line from the center of the lens to the center of the vitreous body. For the refractive indices, the values from the Navarro wide‐angle schematic eye were used: *n* = 1.3777, 1.3391, 1.4222, and 1.3377 for the cornea, aqueous humor, lens, and vitreous humor, respectively.^32^ For each eye model, light rays were traced up to a camera angle of 85°. An example of a subject‐specific eye model is shown in Figure 2a, and the full method to generate such an eye model and to perform the optical simulations has been made publicly available at ZOSPy's GitHub repository.^29^ A representative example of a subject‐specific eye model and subsequent analysis in OpticStudio is included in Supplement A.

**TABLE 1:** Geometric parameters used in the personalized eye models.

	Source	Median	Range
**Axial length [mm]**	Biometry	23.41	22.44–26.39
**Feet‐head retinal radius [mm]**	MRI	10.38	8.96–14.30
**Anterior‐posterior retinal radius [mm]**	MRI	11.49	10.95–12.43
**Spherical equivalent [D]**	Refractometry	−0.88	−6.88–0.25
**Cornea thickness [mm]**	Biometry	0.55	0.47–0.59
**Anterior chamber depth [mm]**	Biometry	3.18	2.79–3.77
**Lens thickness [mm]**	Biometry	3.56	3.17–4.19
**Cornea front curvature [mm]**	Pentacam	7.76	7.23–8.13
**Cornea front asphericity**	Pentacam	0.49	0.14–0.74
**Cornea back asphericity**	Pentacam	0.3	−0.12–0.61
**Lens back curvature [mm]**	Refractometry	4.9	3.95–6.00
**Second nodal point location [mm]**	Calculated	7.4	7.1–7.8

*Note*: The parameters were measured using corneal topography, biometry, refractometry and MRI. The second nodal point location is defined from the cornea front and calculated from the measured optical parameters. Abbreviation: MRI, magnetic resonance imaging.

**FIGURE 3:**
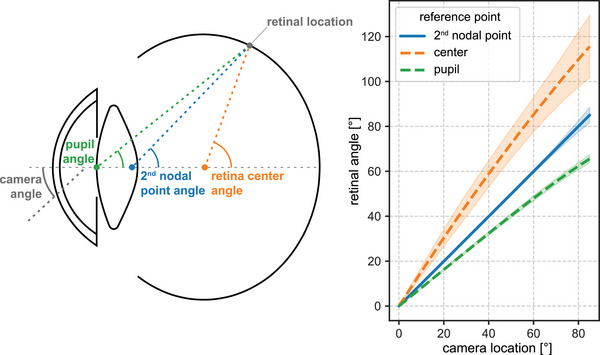
Relation between the camera locations and retinal angles (right) with respect to various reference points (left). For each reference point, the mean and two times the standard deviation are shown. The second nodal point shows the strongest linearity, with small variation between subjects. The second‐order polynomial terms are 7.5 × 10^−5^ for the second nodal point, 2.5 × 10^−3^ for the retinal center, and 8.1 × 10^−4^ for the pupil.

Subsequently, the raytracing results were used to determine a mapping between camera angles and retinal locations. To this end, retinal locations were expressed as angles with respect to a reference location on the optical axis (Figure 3a). Three possible reference points were considered: the center of the retinal ellipse,^25^ the pupil, and the second nodal point.^33^ The second nodal point is a paraxial optical characteristic of the eye, which can be calculated from corneal curvatures, refractometry, and biometry data.^33^, ^34^ For each of the three reference points, a linear approximation of the relation between camera angles and retinal angles was obtained through a fit on the Navarro wide‐angle eye model^32^ for camera angles up to 40°. These mapping methods, which do not require raytracing simulations, were compared with other available methods: a mapping used in the EYEPLAN ocular proton therapy planning system,^13^ a mapping defined by Corcoran et al.,^14^ the Lamberth azimuthal equal‐area projection^12^ and the equidistant polar projection used in the OCTOPUS ocular proton therapy planning system.^15^ In this comparison, the raytracing data is used as “ground truth”. More mathematical details on these methods are provided in Supplement B.

The effects of measurement uncertainties and model assumptions on the mapping method were studied by repeating the simulations with slightly changed eye models. To estimate the impact of errors in the refractometry data on both the location of the second nodal point and the ray tracing results, the impact of an increase of 0.5 D^35^ in the measured spherical equivalent of refraction (SE) was assessed. Furthermore, the effect of differences in retinal shape was modeled by increasing the retinal radius by 0.12 mm, the uncertainty of the MRI‐based retinal shape determination.^31^ The impact of different model refractive indices was studied by repeating the simulations with the refractive indices of the Liou–Brennan model^36^, and the refractive indices for different wavelengths as reported by Escudero‐Sanz et al.^32^ Finally, after correcting for the changed second nodal point location, the second nodal point mapping was applied on a pseudophakic eye by replacing the lens of the Navarro model with a generic intra‐ocular lens model, with a thickness of 1 mm and a refractive index of 1.47.^17^, ^25^ The respective mean differences and standard deviations of these simulations are reported for a camera angle of 40°.

Finally, as the second nodal point reference proved to be the most accurate mapping method, it was implemented in RayOcular (RaySearch Laboratories AB, Stockholm, Sweden, version 2023B), an image‐based ocular proton therapy planning system. To this end, RayOcular's fundus view was configured to have an angular scaling with the nodal point as a reference.^37^, ^38^ A geometrical eye model was defined using the MRI data of a uveal melanoma patient, who provided written informed consent for the use of his clinical data. A neuroradiologist with over 20 years of experience (BV) and an ophthalmic imaging expert with 12 years of experience (JWB) jointly delineated the tumor on the MR images. The tumors were drawn on a contrast‐enhanced T1‐weighted sequence, with an isotropic reconstructed image resolution of 0.3 mm,^4^, ^9^, ^10^ but information of other sequences, in particular native T1, T2, diffusion, and perfusion‐weighted images, was included in the interpretation. Subsequently, the fundus view in RayOcular was configured to plot the intersection between the MRI‐based tumor contour and the retina, using the second nodal point method. Finally, in this fundus view the panoramic fundus photograph was plotted, with its pixel‐to‐degree scaling calculated with PAROS,^17^ while the rotation and translation of the fundus photograph were based on the locations of the fovea and the optic disk. For the part of the tumor visible in the fundus photograph, the distance between the MRI‐based and the fundus‐based contour was calculated.

### 2.1 Statistical analyses

All simulations were performed for camera angles from 0° to 85° in steps of 5°. The simulation results were evaluated in steps of 10°. For the relations between camera angles and retinal angles for different reference points, the means and standard deviations of the retinal locations expressed as angles, are reported across the study population. To quantify the linearity of the relations, second‐order polynomials were fitted, and the second‐order terms were reported. For the comparison between the second nodal point method and alternative mapping methods, the differences are expressed as the Euclidean distances between the predicted and simulated reference retinal locations. For these differences, means and standard deviations are reported as well. Differences are considered significant if there is a difference larger than 0.5 mm in more than 25% of the cohort. Furthermore, the statistical significance of the differences between the absolute errors of different methods at a camera angle of 40° was tested with the one‐sided Wilcoxon signed‐rank test.

## 3 RESULTS

The dataset contained 27 left eyes of healthy subjects with a mean age of 25.6 years (range 18.8–60.9 years). 70% of the subjects were female. The automated optical raytracing simulations succeeded in all 27 subject‐specific eye models; in particular, no vignetting occurred, and the rays reached the retina for all camera angles. Figure 3 shows the relations between camera angles and retinal angles for the three different reference points. The raytracing results show a strong linear relation (*R* ^2^ > 0.99) between camera angles and retinal angles with respect to the second nodal point, with a very small variation (SD = 0.4° at a camera angle of 60°) between subjects. The relations for the other two reference points are slightly nonlinear for angles above 60°: compared with the second nodal point, the second‐order terms for both relations are more than 10 times larger (Figure 3). Furthermore, the retina‐center‐based relation shows a larger variation between subjects. At a camera angle of 60°, for example, the retinal angle with respect to the patient‐specific second nodal point was 59.9° ± 0.4° (mean ± SD), with respect to the retina center 85.2° ± 3.8°, and with respect to the pupil 48.0° ± 0.6°. Data on mean retinal angles and standard deviations for all angles can be found in Table S1. Linear fits on these relations for all simulated angles resulted in slopes of 1.0 for the second nodal point, 1.4 for the retinal center, and 0.8 for the pupil.

**FIGURE 4:**
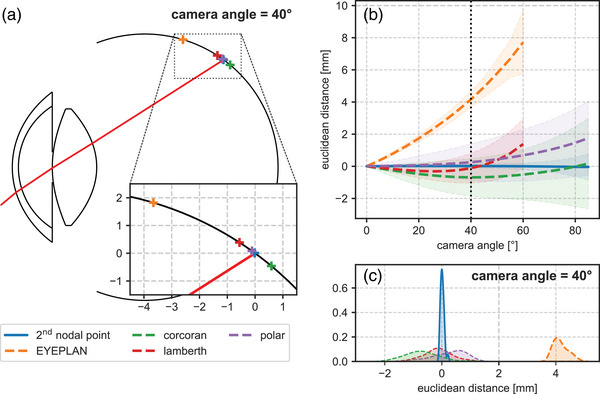
(a) Navarro eye model with a ray trace result for a camera angle of 40°, and typical retinal locations determined using different methods. (b) The corresponding errors are expressed as the Euclidean distance between the true and predicted retinal location. At a camera angle of 40°, the second nodal point method provides the lowest systematic and random errors. The results for the method proposed by Corcoran et al., which expresses retinal locations as an angle with respect to the retina center, show a larger spread. For camera angles larger than 60°, the results of the EYEPLAN and Lamberth projection methods are undefined and have therefore been left out. (c) Kernel density estimations of the difference between the true and predicted retinal locations, at a camera angle of 40°.

Due to the observed linearity and smallest variation between subjects, the second nodal point was considered the most suitable reference point for a non‐raytracing‐based mapping between camera angles and retinal locations. This method was subsequently compared with the approaches used in EYEPLAN,^13^ the mapping defined by Corcoran et al.,^14^ the Lamberth azimuthal equal‐area projection^12^ and the equidistant polar projection.^15^ Figure 4a shows the resulting mappings for the Navarro eye model at a camera angle of 40°. When these results are combined for the whole cohort, the mean (Euclidian distance) error in the retinal location calculated using the second nodal point method was 0.02 mm (SD = 0.06 mm); for EYEPLAN 4.16 mm (SD = 0.25 mm); for Corcoran et al. −0.69 mm (SD = 0.58 mm); for the Lamberth projection −0.12 mm (SD = 0.46 mm) and for the polar projection 0.26 mm (SD = 0.57 mm). Overall, the second nodal point method resulted in the smallest mean error, as well as the smallest standard deviation. This was observed over all camera angles (Figure 4b and Table 2). The absolute error in the retinal location calculated with the second nodal point method was significantly smaller than the absolute errors of all other methods (*p* < 0.001).

**TABLE 2:** Mean Euclidean distances and standard deviations between the “ground‐truth” retinal locations and retinal locations determined from the camera angle using the second nodal point, EYEPLAN, Corcoran, Lamberth projection and polar projection methods.

Camera angle [°]	Second nodal point		EYEPLAN		Corcoran		Lamberth projection		Polar projection	
	Mean	SD	Mean	SD	Mean	SD	Mean	SD	Mean	SD
0	0.00	0.00	0.00	0.00	0.00	0.00	0.00	0.00	0.01	0.00
10	0.01	0.02	**0.79**	**0.05**	−0.26	0.19	−0.17	0.11	0.03	0.14
20	0.02	0.04	**1.73**	**0.11**	−**0.48**	**0.36**	−0.29	0.22	0.07	0.29
30	0.02	0.05	**2.85**	**0.18**	−**0.63**	**0.49**	−**0.30**	**0.33**	0.14	0.43
40	0.02	0.06	**4.16**	**0.25**	−**0.69**	**0.58**	−0.12	0.46	**0.26**	**0.57**
50	0.01	0.08	**5.78**	**0.45**	−**0.65**	**0.67**	**0.38**	**0.61**	**0.43**	**0.69**
60	0.00	0.10	**7.71**	**0.99**	−**0.52**	**0.79**	**1.38**	**0.77**	**0.68**	**0.8**
70	−0.01	0.16			−**0.31**	**0.97**			**1.01**	**0.91**
80	−0.04	0.3			−**0.02**	**1.24**			**1.46**	**1.06**

*Note*: All distances are in mm. Bold values indicate differences larger than 0.5 mm in more than 25% of the subjects. Abbreviation: MRI, magnetic resonance imaging.

Uncertainties in the model parameters, such as refractive indices, and patient‐specific metrics, including retinal shape, had a small impact on the mapping. A 0.5 D higher SE resulted in a shift of 0.002 ± 0.001 mm (mean ± SD) in the location of the second nodal point and a similar negligible difference of 0.009 ± 0.003 mm in the retinal location at a camera angle of 40°. At the same camera angle, a 0.12 mm larger retinal radius resulted in a displacement of 0.09 ± 0.005 mm of the corresponding retinal location. Using the refractive indices of the Liou–Brennan model instead of those reported by Escudero‐Sanz resulted in a 0.08 ± 0.01 mm shift toward the central retina, while the differences between wavelengths of 458 nm and 632.8 nm resulted in a difference of 0.05 mm. Replacing the lens with an IOL resulted in a shift of 0.66 mm in the second nodal point location, and a difference of 0.05 mm between the actual and predicted retinal location. Full data of these simulations are available in Table S2.

**FIGURE 5:**
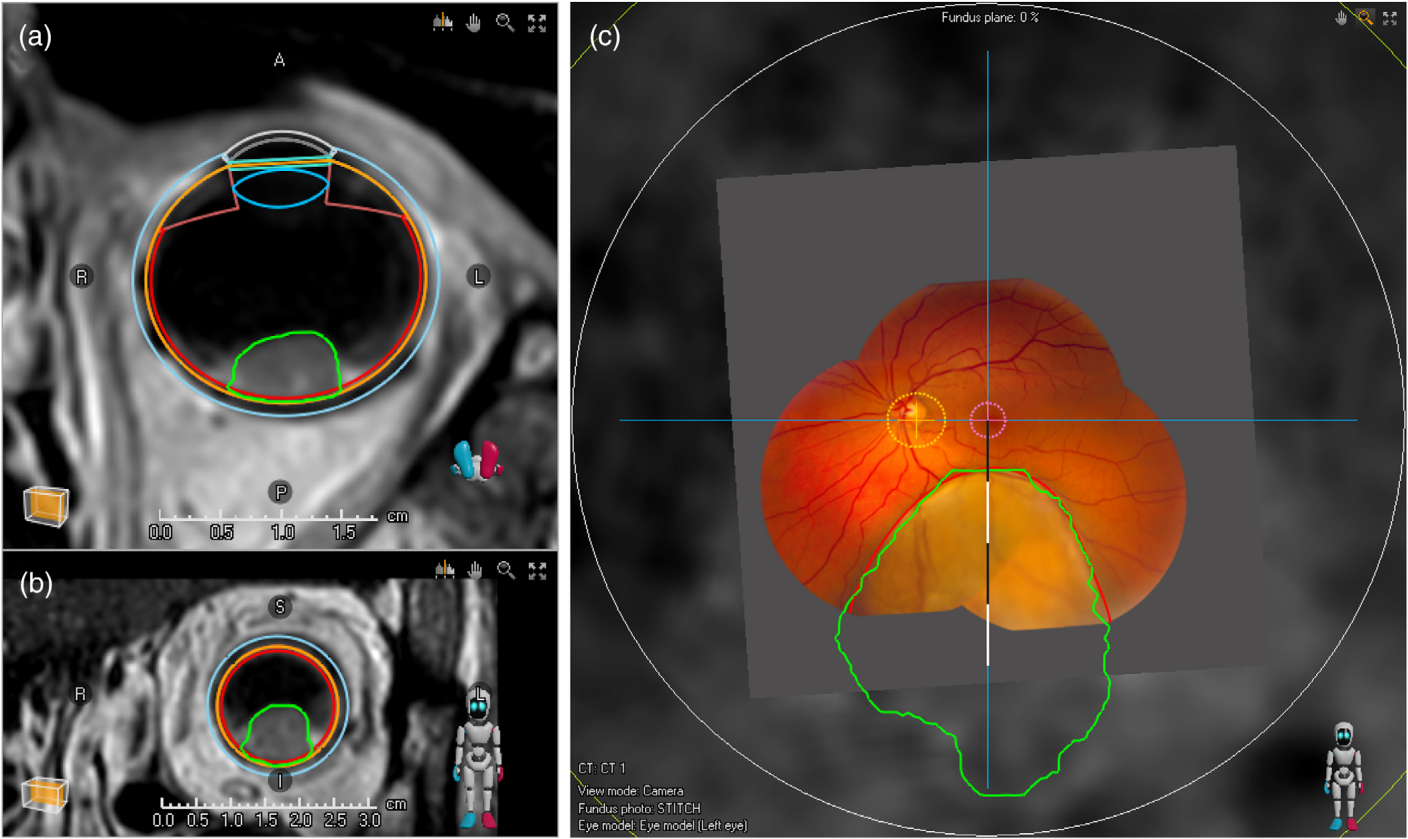
Proof of principle in RayOcular. (a,b) Transversal and coronal sections of the eye model and tumor contour (green), drawn on MRI data. (c) On the fundus projection, the MRI‐based tumor contour (green) overlaps well with the tumor in the fundus photograph (red contour). A single segment of the scale bar corresponds to 10°, so the tumor extends from 8° to 60°, which corresponds to a largest basal diameter of 13 mm. The largest difference is below 1 mm. MRI, magnetic resonance imaging.

For the proof of principle in a clinical treatment planning system, the clinical data of a representative ocular proton therapy patient was used with a centrally located uveal melanoma with a prominence of 5.4 mm (excluding sclera) and a largest basal diameter of 12.3 mm.^5^ This patient was slightly myopic with an objective refraction of −0.25 D and an axial length of 23.4 mm. Using the second nodal point, which was located 7.04 mm behind the anterior corneal surface, as a reference, the tumor extended from 8° to 60°, as shown in Figure 5. The MRI‐based tumor contour corresponds well with the tumor visible in the fundus image, with a median difference of 0.2 mm (IQR = 0.1–0.3 mm). The largest difference of 0.5 mm was located at the edge of the fundus photograph.

## 4 DISCUSSION

The aim of this study was to develop a method to map fundus image locations to the three‐dimensional ocular geometry. Using raytracing simulations on 27 subject‐specific eye models, linear relations were found between camera angles and retinal angles with respect to several reference points. Of the assessed reference points, the second nodal point resulted in the most accurate mapping, with a strong linear relation between camera angles and retinal angles and a small variation between subjects. This observation supports a recent finding by Simpson, who showed a similar linear relation between input and image angles in an idealized schematic eye model.^33^ This study confirms that this relation is also accurate in real eyes, and thus invariant to the subject‐specific ocular anatomy.

Using the second nodal point as a patient‐specific reference proved to provide the most accurate method to determine, without raytracing, the corresponding retinal locations with errors below 0.45 mm up to camera angles of 80°. For the central retina, however, both the Lamberth and polar projection methods also provided a relatively accurate mapping, with small mean errors and inter‐subject variations below 0.3 mm. The accuracy of these methods can largely be explained by the limited refraction of the light rays at these small camera angles, as they intersect the ocular surfaces approximately perpendicularly. At larger camera angles, however, refraction starts to limit the accuracy of these methods as they neglect the eye's optics, which is especially problematic at the cornea‐air interface. Additionally, differences in retinal asphericity contribute to the observed inaccuracies of the Lamberth projection, as this model assumes a spherical retina. When the patient‐specific optics are incorporated by correcting for the central magnification, the inter‐subject variations are significantly reduced (Figure S1). However, a considerable mean error of 0.7 mm at a camera angle of 40° remains present.

The mapping method proposed by Corcoran et al., which forms the basis for the mapping used by the Optos camera,^14^ provides a stable accuracy for the studied camera angles, with mean errors in the order of 0.6 mm and absolute errors smaller than 1 mm at a camera angle of 40° for 67% of the subjects. Conceptually, this method largely resembles the described simulations when retinal locations are expressed with the retinal center as the reference point. As a result, a similar variation in accuracy between subjects is found, which can be largely attributed to the variation in eye length between subjects, which directly relates to the location of the used reference point. The small mean error is likely attributed to the Navarro eye model, which was used to determine the model's parameters.^32^ This eye model was designed to replicate the subject's aberrations, but not necessarily other optical characteristics such as the image location.

The rationale behind the mapping used in EYEPLAN, a widely used ocular proton therapy planning system, is unfortunately poorly documented.^13^ This method uses a reference point located 3.5 mm behind the cornea, which does not directly relate to any optical or anatomical property of the eye. When one would use this specific point as a reference to describe retinal locations, the retinal angle should be smaller than the camera angle, similar to when the pupil would be used as a reference. The optics fit factor, a hyperbolic correction term used in EYEPLAN; however, results in exactly the opposite correction, explaining the large systematic errors. Wulff et al. have empirically shown that the best results are obtained with the optics fit factor set to zero,^37^ which is confirmed by our findings. Nevertheless, in current clinical practice, most of this discrepancy is mitigated by using additional reference fiducials that are sutured adjacent to the tumor.^20^

The second nodal point method was successfully implemented in a clinical therapy planning system and showed a good correspondence between the MRI‐derived tumor contour and fundus photographs. The patient‐specific location of the second nodal point and the corresponding scaling of the fundus photograph could both be calculated using the data from the patient's medical records, showing the clinical feasibility of the method. When these commonly performed ophthalmic measurements are not available, a population value of 7.45 mm can also be used, which will result in a slightly larger variation between subjects of 0.14 mm at a camera angle of 40°. Although many elements of the eye model have been personalized, specific choices in the eye and camera model could impact the accuracy of the performed simulations. Similarly to earlier ophthalmic raytracing studies^21^, ^25^, ^26^, ^27^, ^32^, ^33^ an eye model was used in which all optical elements are centered on a common axis. Although all clinical data was acquired along the visual axis, and the full central 6 mm of the corneal topography was included,^39^ off‐axis properties of the eye, especially lens decentration, and tilt, can affect the simulation's accuracy. Van Vught et al.; however, showed that these deviations only result in minimal, sub‐1‐degree, shifts of retinal illumination locations.^26^, ^40^ A likely more relevant limitation is the use of a rotationally symmetric eye model which therefore ignores astigmatism. The method could be extended to include an orientation‐specific nodal point, but further studies are required to evaluate the clinical relevance of such an extension.^25^, ^41^ Furthermore, population average refractive indices, as reported by Navarro et al.,^32^ were used. Because these values are extensively used in cataract surgery and used to accurately predict a large range of optical parameters of the eye, they can be considered applicable for optical simulations. Moreover, the simulations assessing the impact of various uncertainties of the model show small differences below 0.1 mm, indicating the general robustness of the method. These results also suggest that small medium changes, such as inhomogeneities of the vitreous, will have a limited effect. The second nodal point relation is also valid for pseudophakic eyes for camera angles up to 50°. Future analyses should, however, incorporate more realistic IOL models as their edge design can have a significant impact on peripheral vision.^25^

A second important approximation is the use of an idealized aberration‐free camera. Although the PAROS method corrects for scaling differences in the central part of the fundus image, it does not correct for nonlinearities at the edge of a classical fundus photograph.^17^ These aberrations are likely limited when multiple fundus photographs with a limited field of view are stitched together in a so‐called panoramic fundus image, as shown in Figure 1, but the stitching process itself could introduce additional errors. In addition to this classical method of visualizing the eye's retina, a wide range of fundus camera designs exist, with varying optical properties. For example, scanning laser ophthalmoscopes (SLO), such as the Optos developed by Corcoran et al., can visualize 200° of the retina, but these images show stronger peripheral deformations than classical fundus cameras.^1^, ^19^ A special case is contact fundus cameras, such as the Panoret,^12^ where a gel is applied between the camera and the cornea. As this almost completely cancels refraction at the anterior corneal surface, the described second nodal point relation becomes inaccurate, but additional raytracing simulations show that a similar linear relation can be obtained when the presence of liquid in front of the cornea is included in the calculation of the second nodal point (Supplement C).

Although we expect that these limitations will have a minor impact on the accuracy of the relation between fundus image locations and t 3D imaging, especially compared to the relatively large uncertainties and errors in the current methods,^1^, ^10^ an independent validation of this method is critical. For such a validation study the tantalum fiducials used in ocular proton therapy could be used, as their locations are known on fundus photographs as well as MR images.^2^, ^11^ Such a study should quantify the angle‐dependent uncertainty in fundoscopy‐based contours and determine its effect on the treatment volume.

The proposed fundus image mapping method has several clinical applications. For example, it allows for a more prominent role for fundus images in the ocular proton therapy planning process. A similar mapping can be used for other high‐resolution optical imaging methods, such as Optical Coherence Tomography (OCT) and SLO, to further improve the delineation of the tumor base. Knowledge about the locations of flat tumor extensions and radiation‐sensitive structures such as the macula, which are not clearly visualized in other modalities, helps to accurately plan a dose distribution with optimal vision sparing. This increased accuracy in imaging is an important step to enable a clipless treatment planning procedure, which would eliminate the need for surgery and significantly reduce the complexity of the treatment planning process. Finally, radiation‐induced damage to the retina resulting from radiotherapy can be studied on fundus photographs.^42^, ^43^ Through this mapping method the observed reaction can be correlated to the planned dose distribution, which could aid in the development of Normal Tissue Complication Probability (NTCP) models for the eye.

In this study, a method was developed to accurately map fundus images onto three‐dimensional ocular images, with sub‐millimeter errors. The developed method does not require raytracing simulations and depends only on clinical ophthalmic data, which facilitates its clinical adoption. The method was shown to be more accurate than currently available methods and can be readily applied in radiotherapy treatment planning systems, where it can potentially contribute to a more conformal treatment.

## CONFLICT OF INTEREST STATEMENT

Our institute receives research support from Philips Healthcare and RaySearch Laboratories.

## Supporting information



Supplement A1

Supplement A2

Supplement B

Supplement C

Table S1

Table S2

Figure S1: Difference between true retinal locations and retinal locations calculated with the Lamberth and polar projection methods and the second nodal point method. **A)** Results for the projection methods as described in literature. At a camera angle of 40°, the mean differences are for the Lamberth projection ‐0.12 mm (SD = 0.46 mm) and for the polar projection 0.26 mm (SD = 0.57 mm). **B)** Results for the projection methods when these are corrected for the patient‐specific paraxial magnification. At a camera angle of 40°, the mean errors are for the Lamberth projection 0.68 mm (SD = 0.06 mm) and for the polar projection 0.14 mm (SD = 0.03 mm).
