# Supplementary material for: Patient‐specific mapping of fundus photographs to three‐dimensional ocular imaging
Source: Med Phys. 2024 Dec 12;52(4):2330–9. doi: 10.1002/mp.17576 (PMC11972038; doi:10.1002/mp.17576)
Supplement: Supplementary file 1 — Supplement A1 [file MP-52-2330-s004.pdf]

Scaling peripheral fundus images using a reference point method - Part 1: Raytracing

This notebook illustrates the ray tracing simulations used in the paper *Patient-specific mapping of fundus photographs to three-dimensional ocular imaging* on an eye model based on clinical data.

[1]:
```
import json
import matplotlib.pyplot as plt
import numpy as np
import pandas as pd
import seaborn as sns
import zospy as zp

from helpers import get_nodal_points, get_retina_locations, InputOutputAngles
```

Initialize OpticStudio

Establishing a connection with OpticStudio through the ZOSPy library.

In this example we connect with OpticStudio in `extension` mode. For more extensive simulations we advise to use the `standalone` for a significant increase in computation performace.

[2]:
```
zos = zp.ZOS()
zos.wakeup()
```

[3]:
```
zos.connect_as_extension()
oss = zos.get_primary_system()
```

Define the eye model

The used eye model is based upon clinical measurements of healthy subject.

[4]:
```
# navarro_geometry = {
#     "axial_length": 23.9203,  # mm
#     "cornea_thickness": 0.5,  # mm
#     "anterior_chamber_depth": 3.05,  # mm
#     "lens_thickness": 4.0,  # mm
#     "cornea_front_curvature": 7.72,  # mm
#     "cornea_front_asphericity": -0.26,
#     "cornea_back_curvature": 6.5,  # mm
#     "cornea_back_asphericity": 0,
#     "iris_radius": 0.5,  # mm
#     "lens_front_curvature": 10.2,  # mm
#     "lens_front_asphericity": -3.1316,
#     "lens_back_curvature": -6.0,  # mm
#     "lens_back_asphericity": -1,
#     "retina_curvature": -12.0,  # mm
#     "retina_asphericity": 0.0,
```

```python
# }

geometry = {
    "axial_length": 24.305,  # mm
    "cornea_thickness": 0.5615,  # mm
    "anterior_chamber_depth": 3.345,  # mm
    "lens_thickness": 3.17,  # mm
    "cornea_front_curvature": 7.6967,  # mm
    "cornea_front_asphericity": -0.2304,
    "cornea_back_curvature": 6.2343,  # mm
    "cornea_back_asphericity": -0.1444,
    "iris_radius": 0.5,  # mm
    "lens_front_curvature": 10.2,  # mm
    "lens_front_asphericity": -3.1316,
    "lens_back_curvature": -5.4537,  # mm
    "lens_back_asphericity": -4.1655,
    "retina_curvature": -11.3357,  # mm
    "retina_asphericity": -0.0631,
}

geometry["vitreous_thickness"] = geometry["axial_length"] - (
    geometry["cornea_thickness"]
    + geometry["anterior_chamber_depth"]
    + geometry["lens_thickness"]
)
geometry["retina_radius_z"] = abs(
    geometry["retina_curvature"] / (geometry["retina_asphericity"] + 1)
)
geometry["retina_radius_y"] = abs(
    geometry["retina_curvature"] / np.sqrt(geometry["retina_asphericity"] + 1)
)

# For the Lamberth projection, a spherical retina needs to be used
# mean_retina_radius = np.mean([geometry["retina_radius_z"],␣
 ↪geometry["retina_radius_y"]])
# geometry["retina_curvature"] = geometry["retina_radius_y"] = geometry[
#     "retina_radius_z"
# ] = -mean_retina_radius
# geometry["retina_asphericity"] = 0.0

refractive_indices = {  # at 543 nm (green light)
    "cornea": 1.3777,
    "aqueous": 1.3391,
    "lens": 1.4222,
    "vitreous": 1.3377,
}
```

```python
with open("data/geometry.json", "w") as f:
    json.dump(geometry, f)
```

Initialize the optical system in OpticStudio

For ray tracing, a wavelength of 543 nm (in the center of the visible spectrum) is used, with input beams angles from 0° to 85° in steps of 5°.

Ray aiming (a feature of OpticStudio which shifts peripheral input beams so they pass through the center of the actual pupil) is turned off, as in ophthalmic imaging the entrance pupil is in the center of the image.

[5]:
```python
APERTURE = zp.constants.SystemData.ZemaxApertureType.FloatByStopSize
WAVELENGTH = 0.543  # nm
FIELDS = np.arange(0, 90, 5)  # degrees with respect to the optical axis
```

[6]:
```python
oss.new()

oss.SystemData.Aperture.ApertureType = (
    zp.constants.SystemData.ZemaxApertureType.FloatByStopSize
)
oss.SystemData.Wavelengths.GetWavelength(1).Wavelength = WAVELENGTH
oss.SystemData.RayAiming.RayAiming = zp.constants.SystemData.RayAimingMethod.Off

# Add fields
for i, f in enumerate(np.array(FIELDS).astype(float)):
    if i == 0:
        oss.SystemData.Fields.GetField(1).X = 0
        oss.SystemData.Fields.GetField(1).Y = f
        oss.SystemData.Fields.GetField(1).Weight = 1
    else:
        oss.SystemData.Fields.AddField(X=0, Y=f, Weight=1)
```

Create the eye model

For each of the surfaces the curvature, asphericity, thickness and refractive index is set.

[7]:
```python
# Dummy surface, needed for calculation of input angles
input_beam = oss.LDE.InsertNewSurfaceAt(1)
input_beam.Comment = "Input beam"
input_beam.Thickness = 1.0
input_beam.DrawData.DoNotDrawThisSurface = True

cornea_front = oss.LDE.InsertNewSurfaceAt(2)
cornea_front.Comment = "Cornea Front"
cornea_front.Thickness = geometry["cornea_thickness"]
cornea_front.Radius = geometry["cornea_front_curvature"]
cornea_front.Conic = geometry["cornea_front_asphericity"]
```

```python
zp.solvers.material_model(
    cornea_front.MaterialCell, refractive_index=refractive_indices["cornea"]
)

cornea_back = oss.LDE.InsertNewSurfaceAt(3)
cornea_back.Comment = "Cornea Back / Aqueous"
cornea_back.Thickness = geometry["anterior_chamber_depth"]
cornea_back.Radius = geometry["cornea_back_curvature"]
cornea_back.Conic = geometry["cornea_back_asphericity"]
zp.solvers.material_model(
    cornea_back.MaterialCell, refractive_index=refractive_indices["aqueous"]
)
cornea_back.DrawData.DoNotDrawEdgesFromThisSurface = True

pupil = oss.LDE.GetSurfaceAt(4)
assert pupil.IsStop, "Pupil must be the STOP surface."
pupil.Comment = "Pupil"
pupil.SemiDiameter = geometry["iris_radius"]
zp.solvers.material_model(
    pupil.MaterialCell, refractive_index=refractive_indices["aqueous"]
)
pupil.DrawData.DoNotDrawEdgesFromThisSurface = True

lens_front = oss.LDE.InsertNewSurfaceAt(5)
lens_front.Comment = "Lens Front"
lens_front.Thickness = geometry["lens_thickness"]
lens_front.Radius = geometry["lens_front_curvature"]
lens_front.Conic = geometry["lens_front_asphericity"]
lens_front.SemiDiameter = 4.0  # Larger diameter for visualization purposes
zp.solvers.material_model(
    lens_front.MaterialCell, refractive_index=refractive_indices["lens"]
)

lens_back = oss.LDE.InsertNewSurfaceAt(6)
lens_back.Comment = "Lens Back / Vitreous"
lens_back.Thickness = geometry["vitreous_thickness"]
lens_back.Radius = geometry["lens_back_curvature"]
lens_back.Conic = geometry["lens_back_asphericity"]
zp.solvers.material_model(
    lens_back.MaterialCell, refractive_index=refractive_indices["vitreous"]
)
lens_back.DrawData.DoNotDrawEdgesFromThisSurface = True

retina = oss.LDE.GetSurfaceAt(7)
assert retina.IsImage, "Retina must be the IMAGE surface."
retina.Comment = "Retina"
retina.Radius = geometry["retina_curvature"]
```

```
retina.Conic = geometry["retina_asphericity"]
retina.Thickness = 0
# Set the refractive index of the retina to the vitreous to prevent reflections
zp.solvers.material_model(
    retina.MaterialCell, refractive_index=refractive_indices["vitreous"]
)

# Modify the settings for the visualization of the system
for i in range(1, oss.LDE.NumberOfSurfaces + 1):
    oss.LDE.GetSurfaceAt(
        i
    ).DrawData.DrawEdgesAs = zp.constants.Editors.LDE.SurfaceEdgeDraw.Flat
```

Display the eye model in OpticStudio.

[8]:
```
zp.analyses.systemviewers.viewer_3d(oss)
```

[8]:
```
'AnalysisType': 'Draw3D'
'Data': None
'HeaderData': []
'Messages': []
'MetaData': AnalysisMetadata(DateTime=datetime.datetime(2024, 7, 25, 9, 47, 15,
937000), FeatureDescription='', LensFile='D:\\Zemax\\SAMPLES\\LENS.zmx',
LensTitle='')
'Settings': None
```

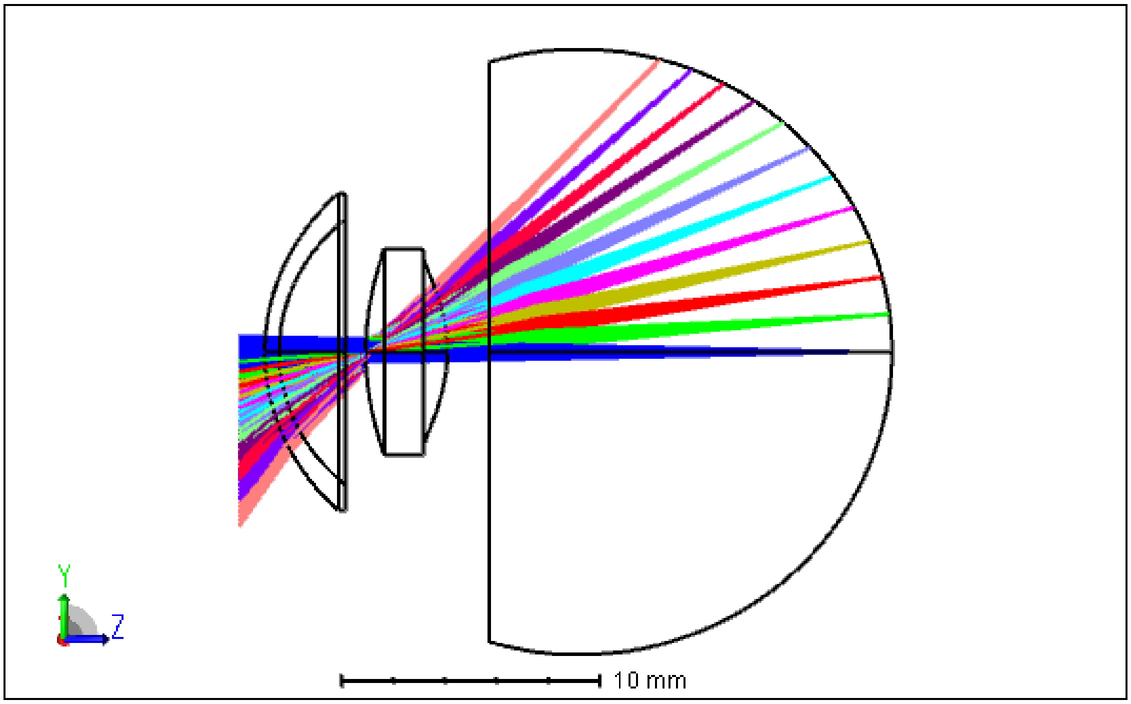

Perform ray tracing

Rays are traced from the light source (the camera) to retina, for the different fields that have been configured in OpticStudio.

The results are evaluated in terms of camera angles and retinal angles. For the latter three reference points are compared: the second nodal point, the retinal center and the pupil.

[9]:
```
ray_trace_results = {}
input_output_angles = []

# Nodal points are calculated in OpticStudio, but can also be calculated␣
↪analytically
np1, np2 = get_nodal_points(oss)

for i in range(1, oss.SystemData.Fields.NumberOfFields + 1):
    y_angle = oss.SystemData.Fields.GetField(i).Y
    ray_trace_results[y_angle] = zp.analyses.raysandspots.single_ray_trace(
        oss, px=0, py=0, field=i, global_coordinates=True
    )
    ray_trace_results[y_angle].Data.RealRayTraceData["InputAngle"] = y_angle

    input_output_angles.append(
        InputOutputAngles.from_ray_trace_result(
            ray_trace_results[y_angle],
            y_angle,
            np2=np2,
            np2_navarro=np2,
            retina_center=(
                geometry["lens_thickness"]
                + geometry["vitreous_thickness"]
                - (
                    abs(
                        geometry["retina_curvature"]
                        / (geometry["retina_asphericity"] + 1)
                    )
                )
            ),
            patient="Patient1",
        )
    )

real_ray_trace_results = pd.concat(
    r.Data.RealRayTraceData for r in ray_trace_results.values()
)
input_output_angles = pd.DataFrame(input_output_angles)
```

Add the retinal locations to the dataframe

[10]:
```python
input_output_angles["retina_location"] = input_output_angles.apply(
    lambda r: get_retina_locations(r, real_ray_trace_results), axis=1
)

input_output_angles
```

[10]:

	input_angle_field	input_angle_cornea	input_angle_pupil	\
0	0.0	0.0	0.000000	
1	10.0	10.0	8.559338	
2	20.0	20.0	17.125957	
3	30.0	30.0	25.710515	
4	40.0	40.0	34.330320	
5	50.0	50.0	43.012066	
6	60.0	60.0	51.792984	
7	70.0	70.0	60.718906	
8	80.0	80.0	69.838344	
9	5.0	5.0	4.279254	
10	15.0	15.0	12.841190	
11	25.0	25.0	21.415104	
12	35.0	35.0	30.014588	
13	45.0	45.0	38.661366	
14	55.0	55.0	47.387416	
15	65.0	65.0	56.234756	
16	75.0	75.0	65.251528	
17	85.0	85.0	74.484459	

	output_angle_pupil	output_angle_np2	output_angle_retina_center	\
0	0.000000	0.000000	0.000000	
1	8.236620	9.924741	13.887107	
2	16.461583	19.876188	27.752562	
3	24.653892	29.866615	41.532014	
4	32.778578	39.883907	55.092636	
5	40.784670	49.883060	68.229900	
6	48.602608	59.775819	80.686513	
7	56.149161	69.430824	92.200759	
8	63.373162	78.731841	102.612037	
9	4.118911	4.960514	6.944355	
10	12.351573	14.895882	20.825151	
11	20.563653	24.866623	34.659589	
12	28.727470	34.873829	48.350894	
13	36.800334	44.890134	61.729413	
14	44.722252	54.850162	74.560403	
15	52.414799	64.642057	86.575621	
16	59.800635	74.128488	97.546003	
17	66.881087	83.250187	107.415926	

output_angle_navarro_np2   location_np2   location_retina_center   patient   \

```
0                    0.000000     3.457872                      8.299343  Patient1
1                    9.924741     3.457872                      8.299343  Patient1
2                   19.876188     3.457872                      8.299343  Patient1
3                   29.866615     3.457872                      8.299343  Patient1
4                   39.883907     3.457872                      8.299343  Patient1
5                   49.883060     3.457872                      8.299343  Patient1
6                   59.775819     3.457872                      8.299343  Patient1
7                   69.430824     3.457872                      8.299343  Patient1
8                   78.731841     3.457872                      8.299343  Patient1
9                    4.960514     3.457872                      8.299343  Patient1
10                  14.895882     3.457872                      8.299343  Patient1
11                  24.866623     3.457872                      8.299343  Patient1
12                  34.873829     3.457872                      8.299343  Patient1
13                  44.890134     3.457872                      8.299343  Patient1
14                  54.850162     3.457872                      8.299343  Patient1
15                  64.642057     3.457872                      8.299343  Patient1
16                  74.128488     3.457872                      8.299343  Patient1
17                  83.250187     3.457872                      8.299343  Patient1

                  retina_location
0                   (20.3985, 0.0)
1   (20.022129705, 2.8982969683)
2   (18.929358005, 5.5933282836)
3   (17.225415858, 7.9060177623)
4   (15.071429925, 9.7049004243)
5   (12.661808084, 10.923468966)
6   (10.196207912, 11.566389056)
7   (7.8495996064, 11.703115207)
8   (5.7383659648, 11.445857555)
9   (20.303833183, 1.4621330149)
10  (19.560230904, 4.2832673229)
11  (18.144798235, 6.8070476922)
12  (16.192992149, 8.8754977161)
13  (13.885875539, 10.388088262)
14  (11.424123782, 11.313897756)
15  (8.9989454527, 11.691614612)
16  (6.7605642505, 11.616108718)
17  (4.7841584997, 11.206051257)
```

Save the output

[11]:
```
real_ray_trace_results.to_csv("data/ray_trace_results.csv", index=False)
input_output_angles.to_csv("data/input_output_angles.csv", index=False)
```

Plot the relations between input angles (camera angles) and output angles (retinal angles)

[12]:

```python
fig, ax = plt.subplots()

sns.lineplot(
    data=input_output_angles,
    x="input_angle_field",
    y="output_angle_np2",
    label="$2^{\\mathrm{nd}}$ nodal point",
)
sns.lineplot(
    data=input_output_angles,
    x="input_angle_field",
    y="output_angle_retina_center",
    label="Retina center",
)
sns.lineplot(
    data=input_output_angles,
    x="input_angle_field",
    y="output_angle_pupil",
    label="Pupil",
)

ax.set_xlabel("Camera angle [°]")
ax.set_ylabel("Retina angle [°]")
ax.set_aspect("equal")
ax.grid()
```

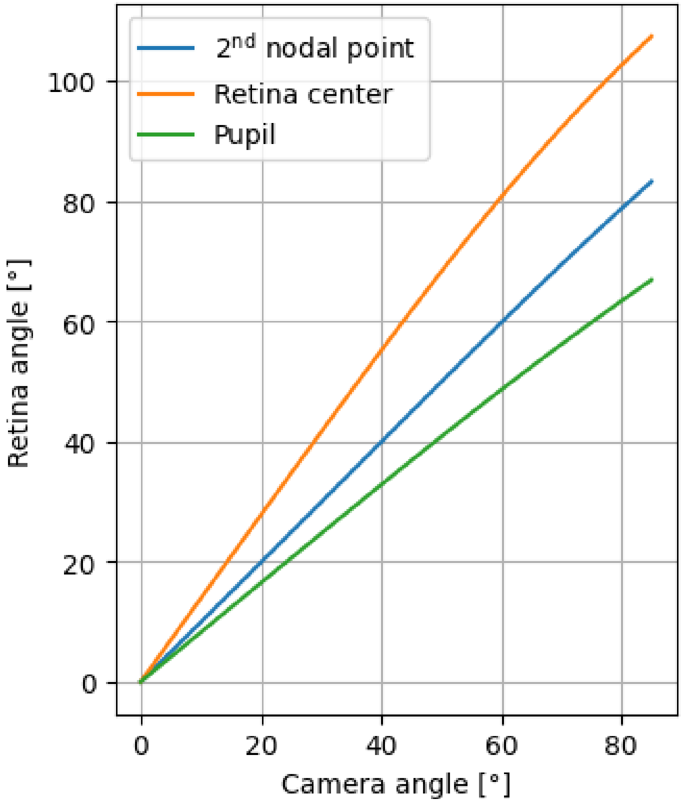
2nd nodal point
Retina center
Pupil
Retina angle [°]
Camera angle [°]
0
20
40
60
80
100
