# Supplementary material for: Patient‐specific mapping of fundus photographs to three‐dimensional ocular imaging
Source: Med Phys. 2024 Dec 12;52(4):2330–9. doi: 10.1002/mp.17576 (PMC11972038; doi:10.1002/mp.17576)
Supplement: Supplementary file 2 — Supplement A2 [file MP-52-2330-s001.pdf]

Scaling peripheral fundus images using a reference point method - Part 2: Analyses

This notebook illustrates the analysis of ray tracing simulations used in the paper *Patient-specific mapping of fundus photographs to three-dimensional ocular imaging*, and compares the proposed method with other fundus mapping methods.

[1]:
```
import json
import matplotlib.pyplot as plt
import numpy as np
import pandas as pd
import seaborn as sns

from scipy.optimize import curve_fit

from helpers import (
    find_ellipse_intersection,
    euclidean_distance,
    _upper_ellipse,
    ellipse_arc_length,
)
```

Load data of the subjected generated by raytracing.ipynb. In addition, reference data of the Navarro eye model are loaded.

[3]:
```
with open("data/navarro_geometry.json") as f:
    navarro_geometry = json.load(f)

with open("data/geometry.json") as f:
    patient_geometry = json.load(f)

with open("data/geometry_lamberth.json") as f:
    patient_geometry_lamberth = json.load(f)

navarro_ray_trace_data = pd.read_csv("data/navarro_ray_trace_results.csv")
navarro_input_output_angles = pd.read_csv("data/navarro_input_output_angles.
 ↪csv")

ray_trace_data = pd.read_csv("data/ray_trace_results.csv")
ray_trace_data_lamberth = pd.read_csv("data/ray_trace_results_lamberth.csv")
input_output_angles = pd.read_csv("data/input_output_angles.csv")
input_output_angles_lamberth = pd.read_csv("data/input_output_angles_lamberth.
 ↪csv")


# Parse tuples in the retina_location column
for df in [
```

```
    navarro_input_output_angles,
    input_output_angles,
    input_output_angles_lamberth,
]:
    df.retina_location = df.retina_location.apply(eval)
```

Plot the relations between camera angles and retinal angles for both the Navarro eye model (solid line) and the patient-specific eye model (dashed line).

[4]:
```
from matplotlib.lines import Line2D

fig, ax = plt.subplots()

for df, ls in zip([navarro_input_output_angles, input_output_angles], ["-",␣
 ↪"--"]):
    sns.lineplot(
        data=df,
        x="input_angle_field",
        y="output_angle_np2",
        ls=ls,
        color="tab:blue",
        label="$2^{\\mathrm{nd}}$ nodal point",
    )
    sns.lineplot(
        data=df,
        x="input_angle_field",
        y="output_angle_retina_center",
        ls=ls,
        color="tab:orange",
        label="Retina center",
    )
    sns.lineplot(
        data=df,
        x="input_angle_field",
        y="output_angle_pupil",
        ls=ls,
        color="tab:green",
        label="Pupil",
    )

# Edit legend
handles = ax.get_legend_handles_labels()[0][:3]
handles += [
    Line2D([], [], linestyle="-", color="black", label="Navarro"),
    Line2D([], [], linestyle="--", color="black", label="Patient"),
]
ax.legend(handles=handles, loc="upper left")
```

```
ax.set_xlabel("Camera angle [°]")
ax.set_ylabel("Retina angle [°]")
ax.set_aspect("equal")
ax.grid()
```

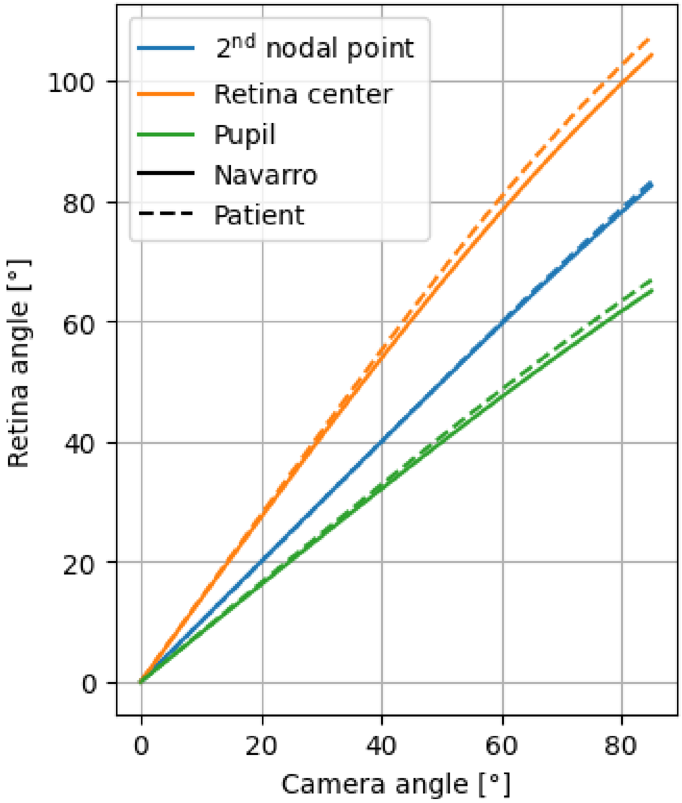

Fit relations between camera angles and retinal angles

Fit linear relations between camera angles and retinal angles for the different reference points, on ray trace data from the Navarro eye. These fits are then used as a reference for the non-ractracing method to predict retinal angles from camera angles. Fits are performed on camera angles up to 40°, due to the observed nonlinearity above 40° for the retinal center and pupil.

[5]:
```
# Fit nodal point method on Navarro data
fit_input_output_angles = navarro_input_output_angles.query("input_angle_field␣
 ↪<= 40")

(c1_np2,), _ = curve_fit(
    lambda theta, c1: c1 * theta,
    xdata=fit_input_output_angles.input_angle_field,
    ydata=fit_input_output_angles.output_angle_np2,
```

```
    p0=1,
)

(c1_retina_center,), _ = curve_fit(
    lambda theta, c1: c1 * theta,
    xdata=fit_input_output_angles.input_angle_field,
    ydata=fit_input_output_angles.output_angle_retina_center,
    p0=1,
)

(c1_pupil,), _ = curve_fit(
    lambda theta, c1: c1 * theta,
    xdata=fit_input_output_angles.input_angle_field,
    ydata=fit_input_output_angles.output_angle_pupil,
    p0=1,
)

print(f"NP2 fit             : {c1_np2:.3f}")
print(f"Retinal center fit : {c1_retina_center:.3f}")
print(f"Pupil fit           : {c1_pupil:.3f}")
```

```
NP2 fit             : 0.999
Retinal center fit : 1.354
Pupil fit           : 0.804
```

Compare the fitted relations with other methods

Use the fitted relations obtained with the Navarro eye model, to determine the corresponding retinal location of the simulated subject without ray tracing. The earlier obtained ray tracing data of this subject are used as a ground truth.

Reference point methods

Determine the retinal location using one of the three reference points (second nodal point, retina center and pupil)

[6]:
```
input_output_angles["output_angle_np2_fit"] = (
    c1_np2 * input_output_angles.input_angle_field
)
input_output_angles["output_angle_retina_center_fit"] = (
    c1_retina_center * input_output_angles.input_angle_field
)
input_output_angles["output_angle_pupil_fit"] = (
    c1_pupil * input_output_angles.input_angle_field
)

# Calculate retinal locations
input_output_angles["retina_location_np2"] = [
```

```
    find_ellipse_intersection(
        r.location_np2,
        np.deg2rad(r.output_angle_np2_fit),
        patient_geometry["retina_radius_z"],
        patient_geometry["retina_radius_y"],
        r.location_retina_center,
    )
    for r in input_output_angles.itertuples()
]

input_output_angles["retina_location_retina_center"] = [
    find_ellipse_intersection(
        r.location_retina_center,
        np.deg2rad(r.output_angle_retina_center_fit),
        patient_geometry["retina_radius_z"],
        patient_geometry["retina_radius_y"],
        r.location_retina_center,
    )
    for r in input_output_angles.itertuples()
]

input_output_angles["retina_location_pupil"] = [
    find_ellipse_intersection(
        0,
        np.deg2rad(r.output_angle_pupil_fit),
        patient_geometry["retina_radius_z"],
        patient_geometry["retina_radius_y"],
        r.location_retina_center,
    )
    for r in input_output_angles.itertuples()
]

input_output_angles["distance_np2"] = euclidean_distance(
    input_output_angles.retina_location, input_output_angles.retina_location_np2
)

input_output_angles["distance_retina_center"] = euclidean_distance(
    input_output_angles.retina_location,
    input_output_angles.retina_location_retina_center,
)

input_output_angles["distance_pupil"] = euclidean_distance(
    input_output_angles.retina_location, input_output_angles.
  ↪retina_location_pupil
)
```

EYEPLAN

Determine the relation between camera angles and retinal angles by the method used in EYEPLAN.

[7]:
```
OPTIC_FIT_FACTOR = 0.126
FIELD_OF_VIEW = 53.4  # degrees
FILM_SIZE = 1  # cm


def inverse_eyeplan_formula(
    camera_angle: float, fov: float = FIELD_OF_VIEW, off: float =␣
 ↪OPTIC_FIT_FACTOR
) -> float:
    """Map `camera_angle` to a retinal angle according to EYEPLAN."""
    return camera_angle * fov / (fov - camera_angle * off)


# EYEPLAN defines a "nodal point" at 3.5 mm behind the cornea
input_output_angles["location_np_eyeplan"] = 3.5 - (
    patient_geometry["cornea_thickness"] +␣
 ↪patient_geometry["anterior_chamber_depth"]
)
input_output_angles["output_angle_eyeplan_formula"] = inverse_eyeplan_formula(
    input_output_angles.input_angle_field
)

# Calculate retinal locations according to EYEPLAN
input_output_angles["retina_location_eyeplan"] = [
    find_ellipse_intersection(
        r.location_np_eyeplan,
        np.deg2rad(r.output_angle_eyeplan_formula),
        patient_geometry["retina_radius_z"],
        patient_geometry["retina_radius_y"],
        r.location_retina_center,
    )
    for r in input_output_angles.itertuples()
]


input_output_angles["distance_eyeplan"] = euclidean_distance(
    input_output_angles.retina_location, input_output_angles.
 ↪retina_location_eyeplan
)
```

Corcoran

Formula proposed by Corcoran et al., used in (early versions of) the Optos ophthalmoscope.

[8]:

```python
def corcoran_formula(
    external_angle: float,
    m: float = 0.819,
    R: float = 12,
    x: float = 3.68,
) -> float:
    """Converts an external angle (camera angle) to an internal angle (retinal␣
↪angle)
    w.r.t. retina center) using the Corcoran formula."""
    external_angle_rad = np.deg2rad(external_angle)

    internal_angle = np.rad2deg(
        m * external_angle_rad
        + 2 * np.arcsin((R - x) / R * np.sin(m * external_angle_rad / 2))
    )

    return internal_angle


input_output_angles["output_angle_corcoran"] = corcoran_formula(
    input_output_angles.input_angle_field
)

# Calculate retinal locations according to Corcoran formula
input_output_angles["retina_location_corcoran"] = [
    find_ellipse_intersection(
        r.location_retina_center,
        np.deg2rad(r.output_angle_corcoran),
        patient_geometry["retina_radius_z"],
        patient_geometry["retina_radius_y"],
        r.location_retina_center,
    )
    for r in input_output_angles.itertuples()
]

input_output_angles["distance_corcoran"] = euclidean_distance(
    input_output_angles.retina_location, input_output_angles.
↪retina_location_corcoran
)
```

The following projection methods work slightly different from the methods evaluated above, as they take cartesian coordinates instead of angles as input. This requires an additional conversion step between input angles and image coordinates. The constants of this conversion are obtained through a fit on ray tracing data of the Navarro eye model.

Lamberth Azimuthal Equal-Area Projection

[9]:
```
def lamberth_image_to_retina_coordinate(
    y_image: float, r: float = 1, z_retina_center: float = 0
) -> tuple[float, float]:
    """
    Convert an image coordinate to a retinal location using the Lamberth␣
 ↪Azimuthal Equal-Area projection.

    Parameters
    ----------
    y_image : float
        Image coordinate.
    r : float
        Radius of the retina. Only spheres are supported.

    Returns
    -------
    tuple[float, float]
        Axial and radial retinal coordinates.
    """
    # Lamberth projection uses coordinates on the unit sphere
    y_retina_norm = np.sqrt(1 - y_image**2 / 4) * y_image
    z_retina_norm = -1 + y_image**2 / 2

    y_retina = y_retina_norm * r
    z_retina = z_retina_norm * r

    # Flip the z-axis: otherwise the back of the retina will get a negative␣
 ↪z-coordinate
    return -1 * z_retina + z_retina_center, y_retina


def lamberth_retina_to_image_coordinate(
    z_retina: float, y_retina: float, r: float = 1, z_retina_center: float = 0
) -> float:
    """
    Convert a retinal location to an image coordinate using the Lamberth␣
 ↪Azimuthal Equal-Area projection.

    Parameters
    ----------
    z_retina : float
        Axial retinal coordinate.
    y_retina : float
        Radial retinal coordinate.
    r : float
```

```
        Radius of the retina. Only spheres are supported.

    Returns
    -------
    float
        Image coordinate.
    """
    y_retina_norm = y_retina / r
    z_retina_norm = (z_retina - z_retina_center) / r

    y_image = np.sqrt(2 / (1 + z_retina_norm)) * y_retina_norm

    return y_image


assert np.isclose(
    y := 0.5,
    lamberth_retina_to_image_coordinate(*lamberth_image_to_retina_coordinate(0.
 ↪5)),
), "Projection roundtrip fails."
```

[10]:

```
def lamberth_angle_conversion_factor(angle: float = 5) -> float:
    """Calculate a scale factor to convert from a camera angle to a Lamberth␣
 ↪projection image coordinate.

    Image coordinates are in 'Lamberth projection space'. The Lamberth␣
 ↪projection is defined on the unit
    sphere, so all projected images have the same size.

    Parameters
    ----------
    angle : float
        Angle for which the ray trace result is used to calculate the␣
 ↪conversion factor.

    Returns
    -------
    float
        Conversion factor in millimeters / degree.
    """
    geometry = navarro_geometry
    ray_trace_data = navarro_ray_trace_data

    mean_retinal_radius = (
        geometry["retina_radius_y"] + geometry["retina_radius_z"]
    ) / 2
    retina_center = geometry["axial_length"] - (
```

```
        geometry["cornea_thickness"]
        + geometry["anterior_chamber_depth"]
        + mean_retinal_radius
    )

    retina_coordinate = ray_trace_data.query(
        "Surf == '7' and InputAngle == @angle"
    ).iloc[0][["Z-coordinate", "Y-coordinate"]]

    image_coordinate = lamberth_retina_to_image_coordinate(
        *retina_coordinate, mean_retinal_radius, retina_center
    )

    return image_coordinate / angle
```

[11]:
```
input_output_angles_lamberth[
    "lamberth_angle_conversion_factor"
] = lamberth_angle_conversion_factor()

input_output_angles_lamberth["lamberth_projected_image_size"] = (
    input_output_angles_lamberth.lamberth_angle_conversion_factor
    * input_output_angles_lamberth.input_angle_field
)

input_output_angles_lamberth[
    "retina_location_lamberth"
] = input_output_angles_lamberth.apply(
    lambda r: lamberth_image_to_retina_coordinate(
        r.lamberth_projected_image_size,
        r=abs(patient_geometry_lamberth["retina_curvature"]),
        z_retina_center=r.location_retina_center,
    ),
    axis=1,
)

input_output_angles_lamberth["distance_lamberth"] = euclidean_distance(
    input_output_angles_lamberth.retina_location,
    input_output_angles_lamberth.retina_location_lamberth,
)

input_output_angles[
    ["distance_lamberth", "retina_location_lamberth"]
] = input_output_angles_lamberth[["distance_lamberth",␣
 ↪"retina_location_lamberth"]]
```

Equidistant polar projection

[12]:
```
from scipy.integrate import quad
from scipy.optimize import minimize_scalar


def octopus_image_to_retina_coordinate(
    y_image: float, geometry: dict[str, float | int]
) -> tuple[float, float]:
    solve_z = minimize_scalar(
        lambda z: abs(
            ellipse_arc_length(
                x1=z,
                x2=geometry["retina_radius_z"],
                r_x=geometry["retina_radius_z"],
                r_y=geometry["retina_radius_y"],
            )
            - y_image
        ),
        bounds=(-geometry["retina_radius_z"], geometry["retina_radius_z"]),
    )

    if not solve_z.success:
        raise RuntimeError(f"Could not solve coordinate for arc length␣
 ↪{y_image=}.")

    z_retina = solve_z.x

    y_retina = _upper_ellipse(
        z_retina,
        r_x=geometry["retina_radius_z"],
        r_y=geometry["retina_radius_y"],
    )

    z_retina_center = (
        geometry["lens_thickness"]
        + geometry["vitreous_thickness"]
        - geometry["retina_radius_z"]
    )
    z_retina += z_retina_center

    return z_retina, y_retina


def octopus_retina_to_image_coordinate(
    z_retina: float, y_retina: float, geometry: dict[str, float | int]
) -> float:
```

```
    z_retina_center = (
        geometry["lens_thickness"]
        + geometry["vitreous_thickness"]
        - geometry["retina_radius_z"]
    )

    z_retina -= z_retina_center

    assert np.isclose(
        _upper_ellipse(
            z_retina,
            r_x=geometry["retina_radius_z"],
            r_y=geometry["retina_radius_y"],
        ),
        y_retina,
    )

    arc_length = ellipse_arc_length(
        z_retina,
        geometry["retina_radius_z"],
        r_x=geometry["retina_radius_z"],
        r_y=geometry["retina_radius_y"],
    )

    return arc_length


assert np.isclose(
    octopus_retina_to_image_coordinate(
        *octopus_image_to_retina_coordinate(2 * np.pi * 12 / 8,␣
 ↪navarro_geometry),
        navarro_geometry,
    ),
    2 * np.pi * 12 / 8,
), "Projection roundtrip fails."
```

[13]:
```
def octopus_angle_conversion_factor(angle: float = 5) -> float:
    """Calculate a scale factor to convert from a camera angle to a Lamberth␣
 ↪projection image coordinate."""
    retina_coordinate = navarro_ray_trace_data.query(
        "Surf == '7' and InputAngle == @angle"
    ).iloc[0][["Z-coordinate", "Y-coordinate"]]

    image_coordinate = octopus_retina_to_image_coordinate(
        *retina_coordinate, navarro_geometry
    )
```

```
    return image_coordinate / angle
```

[14]:
```
input_output_angles["polar_angle_conversion_factor"] =␣
↪octopus_angle_conversion_factor()

input_output_angles["polar_projected_image_size"] = (
    input_output_angles.polar_angle_conversion_factor
    * input_output_angles.input_angle_field
)

input_output_angles["retina_location_polar"] = input_output_angles.apply(
    lambda r: octopus_image_to_retina_coordinate(
        r.polar_projected_image_size,
        geometry=patient_geometry,
    ),
    axis=1,
)

input_output_angles["distance_polar"] = euclidean_distance(
    input_output_angles.retina_location,
    input_output_angles.retina_location_polar,
)

## for debugging: plot the complete list of all angles for all of the methods
# input_output_angles
```

Plot the results

Plot the differences between true (ray tracing) and predicted retinal locations for all methods

[15]:
```
plt.figure()

sns.lineplot(
    input_output_angles,
    x="input_angle_field",
    y="distance_np2",
    label="$2^{\\mathrm{nd}}$ nodal point",
)
sns.lineplot(
    input_output_angles,
    x="input_angle_field",
    y="distance_retina_center",
    label="Retina center",
)
sns.lineplot(
    input_output_angles, x="input_angle_field", y="distance_pupil",␣
↪label="Pupil"
```

```
)
sns.lineplot(
    input_output_angles, x="input_angle_field", y="distance_eyeplan",␣
 ↪label="EYEPLAN"
)
sns.lineplot(
    input_output_angles, x="input_angle_field", y="distance_corcoran",␣
 ↪label="Corcoran"
)
sns.lineplot(
    input_output_angles,
    x="input_angle_field",
    y="distance_lamberth",
    label="Lamberth projection",
)
sns.lineplot(
    input_output_angles,
    x="input_angle_field",
    y="distance_polar",
    label="Polar projection",
)

plt.grid()
plt.xlabel("Camera angle [°]")
plt.ylabel("Euclidean distance [mm]")
```

```
[15]: Text(0, 0.5, 'Euclidean distance [mm]')
```

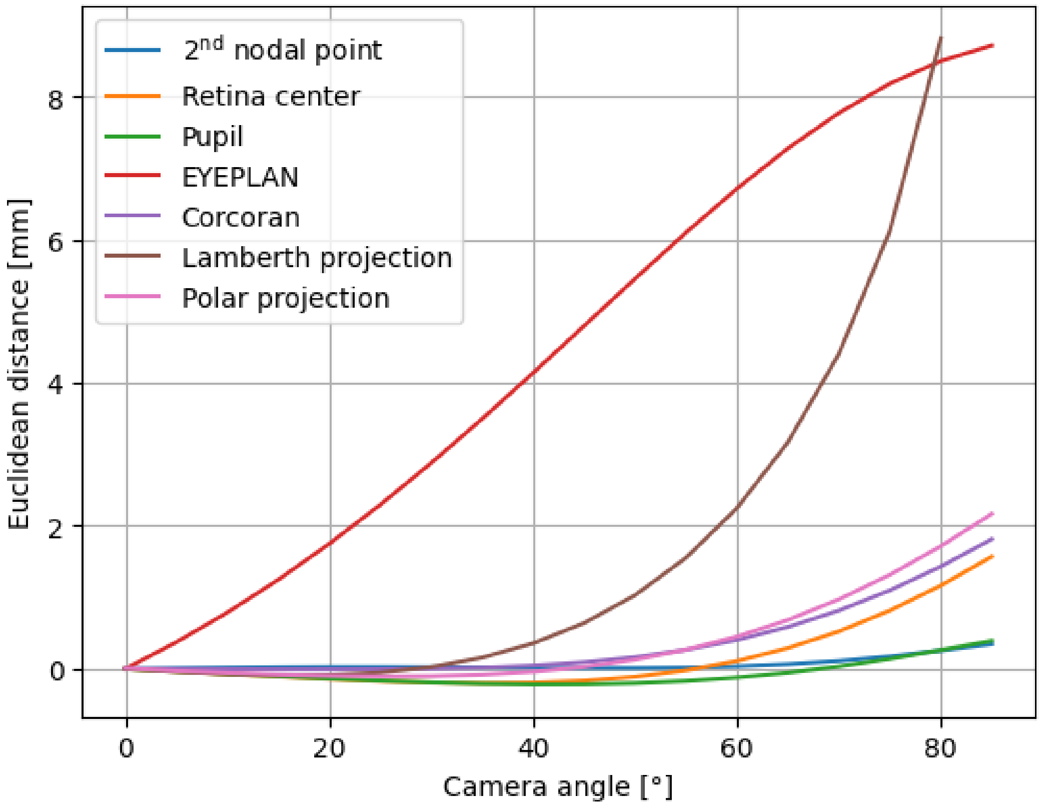

[16]:
```
column_names = {
    "input_angle_field": ("", "Camera angle [°]"),
    "retina_location": ("", "Retina location"),
    "retina_location_np2": ("2nd nodal point", "Retina location"),
    "distance_np2": ("2nd nodal point", "Difference [mm]"),
    "retina_location_retina_center": ("Retina center", "Difference [mm]"),
    "distance_retina_center": ("Retina center", "Difference [mm]"),
    "retina_location_pupil": ("Pupil", "Retina location"),
    "distance_pupil": ("Pupil", "Difference [mm]"),
    "retina_location_eyeplan": ("EYEPLAN", "Retina location"),
    "distance_eyeplan": ("EYEPLAN", "Difference [mm]"),
    "retina_location_corcoran": ("Corcoran", "Retina location"),
    "distance_corcoran": ("Corcoran", "Difference [mm]"),
    "retina_location_lamberth": ("Lamberth", "Retina location"),
    "distance_lamberth": ("Lamberth", "Difference [mm]"),
    "retina_location_polar": ("Polar", "Retina location"),
    "distance_polar": ("Polar", "Difference [mm]"),
}

table = input_output_angles[column_names.keys()]
table.columns = pd.MultiIndex.from_tuples(column_names.values())
```

```
table.map(lambda x: tuple(round(y, 2) for y in x) if isinstance(x, tuple) else␣
↪x).round(
    decimals=2
)
```

[16]:

		2nd nodal point		\
	Camera angle [°]	Retina location	Retina location	Difference [mm]
0	0.0	(20.4, 0.0)	(20.4, 0)	0.00
1	10.0	(20.02, 2.9)	(20.02, 2.92)	0.02
2	20.0	(18.93, 5.59)	(18.92, 5.62)	0.03
3	30.0	(17.23, 7.91)	(17.21, 7.92)	0.03
4	40.0	(15.07, 9.7)	(15.06, 9.71)	0.02
5	50.0	(12.66, 10.92)	(12.65, 10.93)	0.01
6	60.0	(10.2, 11.57)	(10.16, 11.57)	0.03
7	70.0	(7.85, 11.7)	(7.74, 11.7)	0.11
8	80.0	(5.74, 11.45)	(5.49, 11.39)	0.25
9	5.0	(20.3, 1.46)	(20.3, 1.47)	0.01
10	15.0	(19.56, 4.28)	(19.55, 4.31)	0.02
11	25.0	(18.14, 6.81)	(18.13, 6.83)	0.03
12	35.0	(16.19, 8.88)	(16.18, 8.89)	0.02
13	45.0	(13.89, 10.39)	(13.88, 10.39)	0.01
14	55.0	(11.42, 11.31)	(11.41, 11.32)	0.02
15	65.0	(9.0, 11.69)	(8.94, 11.7)	0.06
16	75.0	(6.76, 11.62)	(6.59, 11.59)	0.17
17	85.0	(4.78, 11.21)	(4.45, 11.1)	0.35

	Retina center		Pupil	\
	Difference [mm]	Difference [mm]	Retina location	Difference [mm]
0	(20.4, 0)	0.00	(20.4, 0)	0.00
1	(20.04, 2.83)	-0.07	(20.04, 2.83)	-0.07
2	(19.0, 5.47)	-0.14	(19.0, 5.47)	-0.14
3	(17.36, 7.77)	-0.19	(17.36, 7.77)	-0.19
4	(15.23, 9.6)	-0.20	(15.25, 9.58)	-0.22
5	(12.77, 10.88)	-0.11	(12.85, 10.85)	-0.20
6	(10.09, 11.58)	0.11	(10.32, 11.55)	-0.12
7	(7.33, 11.67)	0.52	(7.82, 11.7)	0.03
8	(4.61, 11.15)	1.16	(5.48, 11.39)	0.26
9	(20.31, 1.43)	-0.04	(20.31, 1.43)	-0.04
10	(19.6, 4.18)	-0.11	(19.6, 4.19)	-0.11
11	(18.25, 6.67)	-0.17	(18.24, 6.67)	-0.17
12	(16.35, 8.74)	-0.20	(16.35, 8.74)	-0.21
13	(14.04, 10.31)	-0.17	(14.08, 10.29)	-0.22
14	(11.45, 11.31)	-0.02	(11.59, 11.27)	-0.17
15	(8.71, 11.7)	0.29	(9.05, 11.69)	-0.06
16	(5.96, 11.49)	0.81	(6.62, 11.6)	0.14
17	(3.31, 10.67)	1.57	(4.41, 11.09)	0.39

	EYEPLAN Retina location	Difference [mm]	Corcoran Retina location	Difference [mm]
0	(20.4, 0)	0.00	(20.4, 0)	0.00
1	(19.8, 3.65)	0.79	(20.02, 2.89)	-0.00
2	(17.96, 7.05)	1.75	(18.93, 5.59)	-0.01
3	(15.01, 9.74)	2.88	(17.22, 7.91)	0.00
4	(11.28, 11.35)	4.13	(15.03, 9.73)	0.05
5	(7.26, 11.67)	5.45	(12.51, 10.98)	0.16
6	(3.53, 10.76)	6.71	(9.8, 11.62)	0.40
7	(0.56, 9.01)	7.77	(7.04, 11.65)	0.81
8	(-1.46, 6.93)	8.50	(4.36, 11.07)	1.43
9	(20.25, 1.83)	0.37	(20.3, 1.46)	-0.00
10	(19.03, 5.41)	1.24	(19.56, 4.28)	-0.01
11	(16.61, 8.51)	2.29	(18.15, 6.8)	-0.01
12	(13.22, 10.7)	3.49	(16.18, 8.89)	0.02
13	(9.27, 11.67)	4.79	(13.8, 10.43)	0.09
14	(5.33, 11.35)	6.10	(11.17, 11.38)	0.26
15	(1.93, 9.96)	7.27	(8.42, 11.71)	0.58
16	(-0.56, 7.97)	8.18	(5.68, 11.43)	1.10
17	(-2.14, 5.92)	8.71	(3.09, 10.57)	1.81

\

	Lamberth Retina location	Difference [mm]	Polar Retina location	Difference [mm]
0	(20.4, 0.0)	0.00	(20.4, 0.01)	0.01
1	(20.06, 2.83)	-0.07	(20.04, 2.84)	-0.06
2	(19.03, 5.53)	-0.09	(18.98, 5.51)	-0.10
3	(17.33, 7.98)	0.03	(17.3, 7.83)	-0.10
4	(14.94, 10.01)	0.35	(15.11, 9.68)	-0.04
5	(11.87, 11.42)	1.03	(12.54, 10.97)	0.13
6	(8.12, 11.9)	2.25	(9.75, 11.63)	0.45
7	(3.68, 10.89)	4.40	(6.88, 11.63)	0.97
8	(-1.44, 6.57)	8.81	(4.09, 10.98)	1.71
9	(20.31, 1.42)	-0.04	(20.31, 1.43)	-0.03
10	(19.63, 4.21)	-0.09	(19.59, 4.21)	-0.08
11	(18.27, 6.8)	-0.05	(18.21, 6.72)	-0.11
12	(16.22, 9.06)	0.16	(16.26, 8.82)	-0.08
13	(13.49, 10.81)	0.64	(13.86, 10.4)	0.03
14	(10.08, 11.8)	1.55	(11.17, 11.38)	0.27
15	(5.98, 11.64)	3.17	(8.32, 11.71)	0.68
16	(1.21, 9.42)	6.12	(5.47, 11.39)	1.31
17	(-4.25, nan)	NaN	(2.77, 10.42)	2.17
