# Supplementary material for: Patient‐specific mapping of fundus photographs to three‐dimensional ocular imaging
Source: Med Phys. 2024 Dec 12;52(4):2330–9. doi: 10.1002/mp.17576 (PMC11972038; doi:10.1002/mp.17576)
Supplement: Supplementary file 3 — Supplement B [file MP-52-2330-s002.pdf]

Supplement B – Mathematical description of alternative mapping methods

The EYEPLAN ocular proton therapy planning system[13] and the OPTOS fundus mapping camera[14] use different relations to link retinal locations to camera locations, both of which are currently used in clinical practice. Furthermore, Via et al.[12] propose the Lambert azimuthal equal-area projection to map retinal locations to locations on a fundus image. This appendix provides the mathematical description behind these mappings in the context of the comparison shown in Figure 4.

Corcoran

In the thesis of Corcoran[14], which describes the development of the OPTOS fundus camera, the following relation was proposed:

$$\theta_{ret} = \bar{m}\theta_{cam} + 2\sin^{-1}\left(\frac{R-x}{R}\sin\left(\frac{\bar{m}\theta_{cam}}{2}\right)\right),$$

with θ the angle of the retinal location with respect to the retinal center, θ' the camera angle, $\bar{m}$ the ocular magnification from cornea and lens, x the distance between the iris and the camera rotation point, and R the radius of the retina. Although the geometric parameters $\bar{m}, x$ and R are different for each eye, only the use of population-average values is reported, which are $\bar{m}$ = 0.819, x = 3.86 mm and R = 12 mm.

Equidistant polar projection

The OCTOPUS ocular proton therapy planning system maps retinal locations to image locations using the equidistant polar projection.[15] This method projects retinal locations onto a polar coordinate system, where the radial distance equals the arc length from the on-axis location of the retina to the retinal location. A point $P(t)$ on an ellipse with semi-major axis A and semi-minor axis B is defined as $(A\cos t, B\sin t)$. For a point $P(t_0)$ on the ellipse, the arc length $L(t_0)$ between the vertex pole and $P(t_0)$ is given by

$$L(t_0) = A\int_0^{t_0} \sqrt{1-\varepsilon^2\sin^2 t}\, dt$$

with ε the eccentricity of the ellipse. In the polar projection, a point P on the retina with polar angle β and arc length L from the posterior pole is projected to $(r,\phi) = (L,\beta)$ on the fundus image.

EYEPLAN

EYEPLAN maps camera/fundus photograph locations to locations on the 3D retina, by expressing these locations as an angle with respect to a reference point that is located 3.5 mm behind the cornea front, through the following relation:

$$\theta_{cam} = \theta_{ret}\left(\frac{1}{1+OFF\,\frac{\theta_{ret}}{FOV}}\right),$$

with θ_{ret} the angle of the retinal location with respect to the reference point, θ_{cam} the camera angle (or location on the fundus photograph), OFF an optic fit factor and FOV the field of view of the camera. In this study, the EYEPLAN default values for FOV and OFF, respectively 0.126 and 53.4°, were used.

Lamberth azimuthal equal-area projection

Via et al.[12] propose the Lambert azimuthal equal-area projection to map retinal locations to locations on a fundus image. This projection defines a mapping between a three-dimensional coordinate (x, y, x) on the unit sphere and an image coordinate (X_L, Y_L):

$$(X_L, Y_L) = \left(\sqrt{\frac{2}{1+z}} \cdot x, \sqrt{\frac{2}{1+z}} \cdot y\right)$$

For the comparison between mapping methods, retinal coordinates were first normalized to the unit sphere after which the Lambert projection was applied. Because this projection is only defined for a sphere, a spherical retina was assumed with the mean radius of the ellipsoidal retina.

RayOcular

In RayOcular version 2023B both the equidistant polar projection and the EYEPLAN method have been implemented, in which in the latter the mapping parameters, such as OFF and reference point, can modified.[38] By setting the OFF to 0 and the reference point to the location of the second nodal point, the second nodal point method is effectively implemented in RayOcular.
