# Supplementary material for: Patient‐specific mapping of fundus photographs to three‐dimensional ocular imaging
Source: Med Phys. 2024 Dec 12;52(4):2330–9. doi: 10.1002/mp.17576 (PMC11972038; doi:10.1002/mp.17576)
Supplement: Supplementary file 4 — Supplement C [file MP-52-2330-s006.pdf]

Supplement C – Simulation results with a contact camera

To assess the effect of using a contact camera instead of a non-contact camera, the raytracing simulations were repeated with water instead of air in front of the cornea. Simulations were performed with a wavelength of 543 nm and a refractive index of 1.3333 for water. The ray tracing simulations show that the refraction at the cornea is reduced, and thus the second nodal point shifted anteriorly compared to a non-contact camera (Figure 1). Throughout this document, this second nodal point determined with water in front of the cornea will be used.

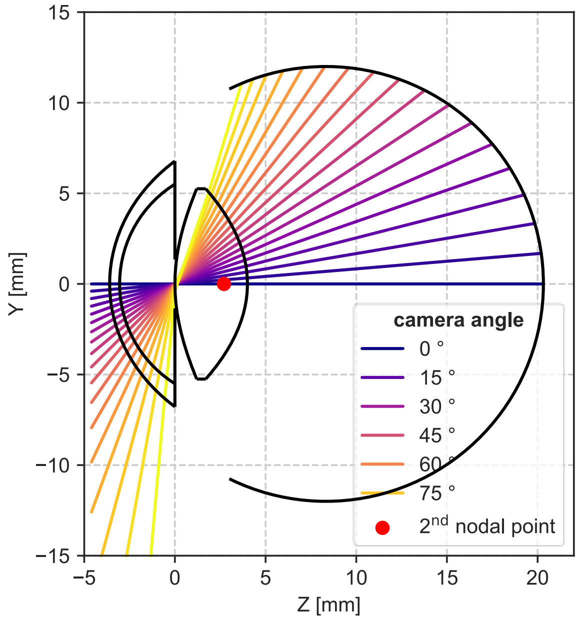

Figure 1: Raytracing result for the Navarro eye model for camera angles up to 85°.

The relations between camera angles and retinal angles with respect to the second nodal point, retinal center, and pupil remain linear, but the slopes are larger than those obtained without water in front of the eye (Figure 2). Similarly to the non-contact camera simulations, the second nodal point shows the strongest linearity. Although the variation between subjects has increased compared to the simulations with a non-contact camera, the variation remains small.

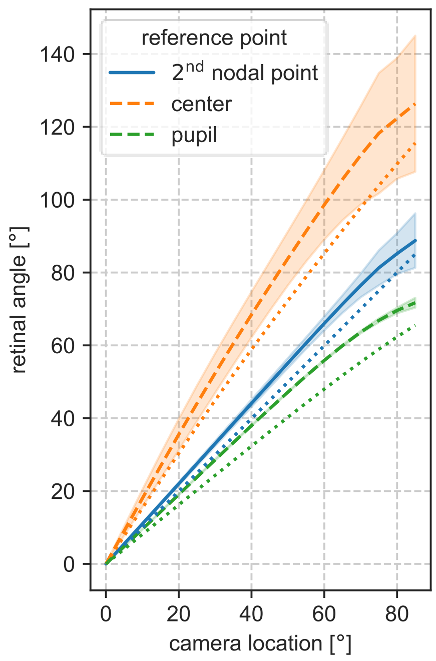

Figure 2: Relations between camera locations and retinal locations for different reference points, with water in front of the cornea. The dotted lines indicate the mean retinal angle for a non-contact camera.

A linear fit was performed on the camera angles and retinal angles with respect to the second nodal point, to determine a mapping between camera angles and retinal locations. Up to camera angles of 60°, the systematic and random errors in the retinal locations calculated with this mapping remain small. For larger camera angles, the errors increase significantly (Figure 3).

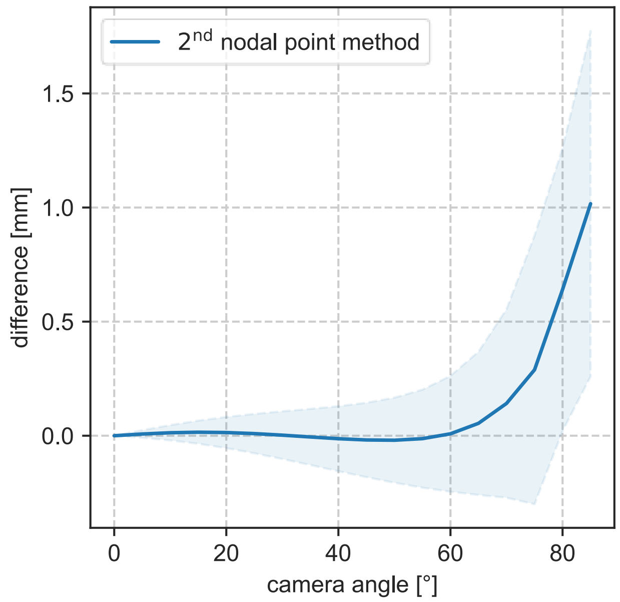

Figure 3: Euclidean distance between the true retinal location and retinal location calculated using the second nodal point method, for a contact camera.
