# Supplementary material for: Patient‐specific mapping of fundus photographs to three‐dimensional ocular imaging
Source: Med Phys. 2024 Dec 12;52(4):2330–9. doi: 10.1002/mp.17576 (PMC11972038; doi:10.1002/mp.17576)
Supplement: Supplementary file 5 — Table S1 [file MP-52-2330-s003.docx]

| **Camera angle [°]** | **Second nodal point** | | **Retina center** | | **Pupil** | |
| --- | --- | --- | --- | --- | --- | --- |
|  | **Mean** | **SD** | **Mean** | **SD** | **Mean** | **SD** |
| 0 | 0 | 0 | 0 | 0 | 0 | 0 |
| 10 | 9.95 | 0.07 | 15.32 | 1.05 | 8.1 | 0.1 |
| 20 | 19.91 | 0.13 | 30.36 | 1.91 | 16.21 | 0.2 |
| 30 | 29.9 | 0.17 | 44.9 | 2.52 | 24.3 | 0.29 |
| 40 | 39.9 | 0.22 | 58.87 | 2.93 | 32.34 | 0.38 |
| 50 | 49.9 | 0.28 | 72.29 | 3.31 | 40.26 | 0.47 |
| 60 | 59.92 | 0.4 | 85.25 | 3.85 | 47.99 | 0.57 |
| 70 | 69.97 | 0.65 | 97.78 | 4.75 | 55.41 | 0.66 |
| 80 | 80.04 | 1.25 | 109.82 | 6.13 | 62.34 | 0.77 |

**Table S-1:** Mean and standard deviation for retinal angles with respect to different reference points.
