# Supplementary material for: Patient‐specific mapping of fundus photographs to three‐dimensional ocular imaging
Source: Med Phys. 2024 Dec 12;52(4):2330–9. doi: 10.1002/mp.17576 (PMC11972038; doi:10.1002/mp.17576)
Supplement: Supplementary file 6 — Table S2 [file MP-52-2330-s005.docx]

| **Camera angle [°]** | **Refraction + 0.5 D [mm]** | | **Retinal radius + 0.12 mm [mm]** | | **Liou-Brennan eye [mm]** | | **Different wavelengths [mm]** | **Pseudophakic eye**  **[mm]** |
| --- | --- | --- | --- | --- | --- | --- | --- | --- |
|  | **Mean** | **SD** | **Mean** | **SD** | **Mean** | **SD** |  |  |
| 0 | 0.00 | 0.00 | 0.00 | 0.00 | 0.00 | 0.00 | 0.00 | 0.00 |
| 5 | 0.00 | 0.00 | 0.00 | 0.00 | 0.00 | 0.00 | 0.01 | 0.00 |
| 10 | 0.01 | 0.00 | 0.01 | 0.00 | -0.01 | 0.00 | 0.01 | 0.00 |
| 15 | 0.01 | 0.00 | 0.01 | 0.00 | -0.01 | 0.00 | 0.02 | 0.00 |
| 20 | 0.01 | 0.00 | 0.02 | 0.00 | -0.02 | 0.00 | 0.03 | 0.01 |
| 25 | 0.01 | 0.00 | 0.04 | 0.00 | -0.03 | 0.00 | 0.03 | 0.01 |
| 30 | 0.01 | 0.00 | 0.05 | 0.00 | -0.05 | 0.01 | 0.04 | 0.02 |
| 35 | 0.01 | 0.00 | 0.07 | 0.01 | -0.06 | 0.01 | 0.05 | 0.02 |
| 40 | 0.01 | 0.00 | 0.09 | 0.01 | -0.08 | 0.01 | 0.05 | 0.03 |
| 45 | 0.01 | 0.00 | 0.10 | 0.01 | -0.11 | 0.01 | 0.06 | 0.05 |
| 50 | 0.01 | 0.01 | 0.12 | 0.01 | -0.14 | 0.02 | 0.06 | 0.06 |
| 55 | 0.01 | 0.01 | 0.13 | 0.01 | -0.17 | 0.02 | 0.07 | 0.09 |
| 60 | 0.01 | 0.01 | 0.15 | 0.01 | -0.21 | 0.02 | 0.07 | 0.13 |
| 65 | 0.01 | 0.01 | 0.15 | 0.01 | -0.27 | 0.03 | 0.08 | 0.18 |
| 70 | 0.01 | 0.01 | 0.16 | 0.01 | -0.34 | 0.04 | 0.08 | 0.26 |
| 75 | 0.00 | 0.01 | 0.16 | 0.01 | -0.43 | 0.04 | 0.08 | 0.39 |
| 80 | 0.00 | 0.01 | 0.15 | 0.01 | -0.55 | 0.05 | 0.08 | 0.63 |
| 85 | 0.00 | 0.01 | 0.14 | 0.01 | -0.72 | 0.06 | 0.08 | 1.09 |

**Table S-2:** Effect of changes in various parameters of the eye model on the retinal locations obtained with ray tracing, as a function of camera angle. Except for the pseudophakic eye, differences are expressed as the Euclidean distance [mm] between the retinal location in the original eye model and the corresponding retinal location in the modified eye model. The effect of chromatic differences has only been simulated on the Navarro schematic eye, with wavelengths of 458 nm and 632.8 nm. The effect of replacing the lens with an IOL has only been studied on the Navarro schematic eye.
