# Supplementary figures and images for: Patient‐specific mapping of fundus photographs to three‐dimensional ocular imaging
Source: Med Phys. 2024 Dec 12;52(4):2330–9. doi: 10.1002/mp.17576 (PMC11972038; doi:10.1002/mp.17576)

A. Uncorrected projections

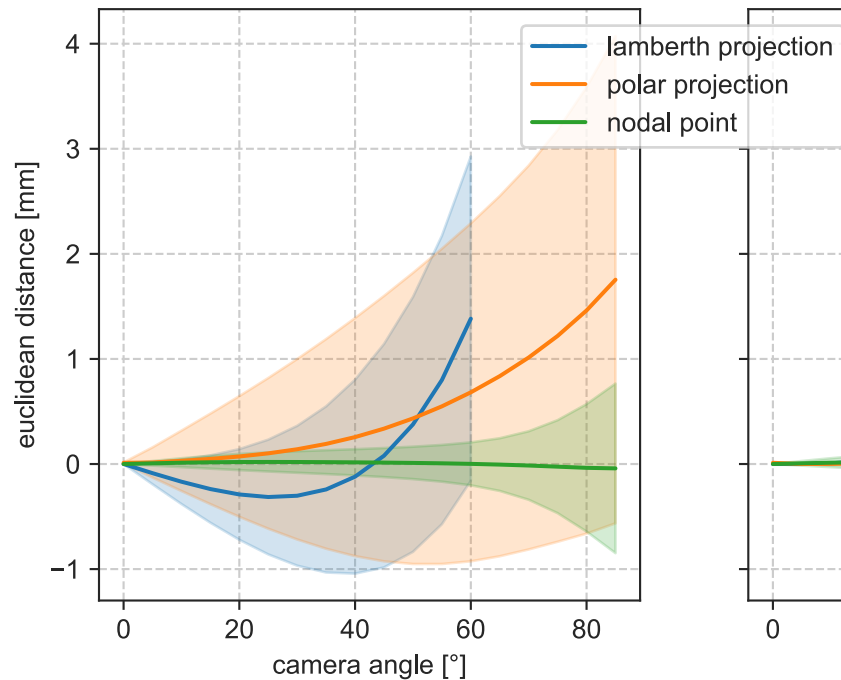

B. Corrected for central magnification

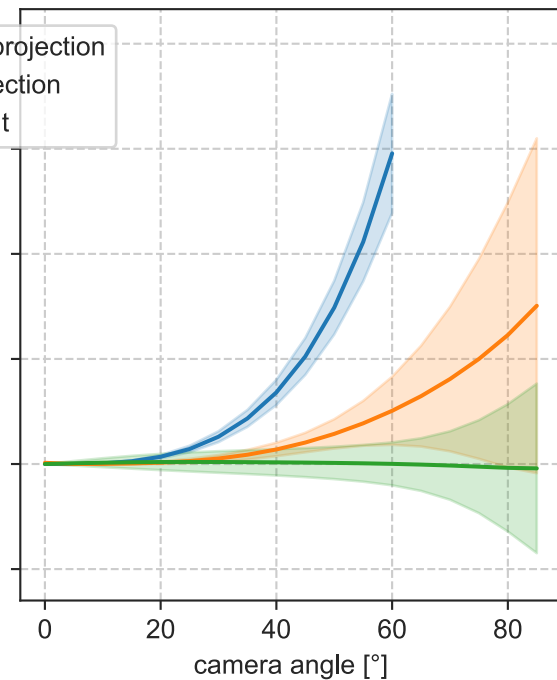

Supplement: Supplementary file 7 — Figure S1: Difference between true retinal locations and retinal locations calculated with the Lamberth and polar projection methods and the second nodal point method. A) Results for the projection methods as described in literature. At a camera angle of 40°, the mean differences are for the Lamberth projection ‐0.12 mm (SD = 0.46 mm) and for the polar projection 0.26 mm (SD = 0.57 mm). B) Results for the projection methods when these are corrected for the patient‐specific paraxial magnification. At a camera angle of 40°, the mean errors are for the Lamberth projection 0.68 mm (SD = 0.06 mm) and for the polar projection 0.14 mm (SD = 0.03 mm). [file MP-52-2330-s007.pdf]
